# Exometabolome variation in the fungal pathogen of humans *Candida albicans* reveals specificities at the genetic clade and strain levels

**DOI:** 10.1128/aem.01512-25

**Published:** 2026-03-18

**Authors:** Leovigildo Rey Alaban, Andrei Bunescu, Joséphine Abi-Ghanem, Frédéric Bequet, Julien Lebrat, Pawel Tulinski, Daria Kosmala, Marie-Elisabeth Bougnoux, Christophe d'Enfert, Vincent Thomas

**Affiliations:** 1 BIOASTER Microbiology Technology Institute 397933 , Lyon, France; 2 Ecole Doctorale Bio Sorbonne Paris Cité (BioSPC), Université Paris Cité 555089, Paris, France; 3 Northern Iloilo State University 316294, Estancia, Iloilo, Philippines; 4 Institut Servier d'Innovation Thérapeutique, Gif-sur-Yvette, France; 5 Unité Biologie et Pathogénicité Fongiques, Institut Pasteur, Université Paris Cité, INRAE USC2019, Inserm U1359 555089, Paris, France; 6 Unité de Parasitologie-Mycologie, Service de Microbiologie clinique, Hôpital Necker-Enfants-Malades, Assistance Publique des Hôpitaux de Paris (APHP) 246596, Paris, France; 7 Danone Global Research & Innovation Centre 72832 , Gif-sur-Yvette, France; Chalmers tekniska hogskola AB, Gothenburg, Sweden

**Keywords:** *Candida albicans*, clade-specific metabolic profile, isolate-specific variation, exometabolome, NMR

## Abstract

*Candida albicans* is a fungal pathobiont that thrives in different host niches and successfully confronts the host immune defenses. The success of *C. albicans* as a pathogen relies on a highly plastic metabolism, with examples of variations across isolates; however, detailed characterization of how *C. albicans* genetic diversity impacts the content of the metabolome is still missing. Here, we have implemented an NMR (nuclear magnetic resonance)-based high-throughput workflow to probe exometabolome variation in *in vitro* cultures of 96 clinical isolates spread across several genetic clusters (clades) that structure the *C. albicans* population. Our workflow allowed for batch analysis, with robust replication (six or more replicates) of the culture supernatants from these isolates. Results of this analysis highlighted the extent of metabolic divergence between clades (clade-specific variation) and within clades (isolate-specific variation). In particular, Clade 13 isolates (corresponding to the *Candida africana* isolates) showed metabolic specificities relative to isolates in other clades, notably non-consumption of trehalose and weak utilization of choline. Isolate-specific variation was mainly driven by metabolites involved in central carbon metabolism.

**IMPORTANCE:**

This study provides one of the most extensive analyses of *Candida albicans* exometabolome— comprising 96 isolates across all major genetic clades. By developing an NMR (nuclear magnetic resonance)-based, high-throughput workflow for the analysis of supernatants of *in vitro* cultured *C. albicans*, we demonstrated the extent of metabolic differences both between clades (clade-specific variation) and among isolates within each clade (isolate-specific variation). Isolates from Clade 13, which corresponds to *Candida africana*, exhibited distinct metabolic profiles compared to isolates from other clades, particularly in their inability to utilize trehalose and their reduced capacity to use choline. Variation among individual isolates was largely attributable to differences in metabolites associated with central carbon metabolism.

## INTRODUCTION

*Candida albicans* is a fungal pathobiont specialized to colonize the human body (1), which is acquired early in life and becomes part of the gastrointestinal and genital microflora of healthy individuals (2). Yet, it is also an opportunistic pathogen, becoming infectious in individuals with a weakened immune system, impaired mucosal barrier, or dysregulated immune responses (3, 4). These infections can be costly to treat and are increasingly non-responsive to treatments, leading to high mortality rates, particularly when acquired in hospitals or healthcare facilities (5, 6).

The ability of *C. albicans* to navigate the host milieu or to engage the host defense is powered by a highly plastic metabolism (7). Metabolic versatility allows *C. albicans* to simultaneously assimilate multiple carbon sources, adapt to host environments, and regulate central carbon metabolism to respond rapidly to stress (7–13). Although much is known about the manifestations of this plasticity, most studies have focused on a few reference isolates, which have been shown to exhibit contrasting metabolic responses in experiments involving host-pathogen interactions (14). Thus, while metabolic variations between isolates are apparent in the literature, the information on the extent of these variations remains fragmented. In particular, data are scarce about the exometabolome—the entire set of small molecules released to the environment—of *C. albicans* and its variations across strains. Knowledge of the exometabolome should provide information on how *C. albicans* obtains nutrients, competes with other microbiome constituents, and engages with the host (15, 16).

Investigating the exometabolome in *C. albicans* is confronted by both biological and technical constraints. First, *C. albicans* shows significant strain diversity at the genomic and phenotypic levels, and as such, strains are routinely organized into genetic clusters or clades via multi-locus sequence typing (MLST) and population genomics. Current nomenclatures based on the MLST of 1,159 isolates and genome sequencing of 182 isolates identify at least 23 clades (Clades 1–18 and A–E) (17, 18), with additional clades proposed, as some isolates (referred to as singletons) do not associate with the defined clades. Clade 13 is unique in that it includes *C. albicans* isolates (otherwise referred to as *Candida africana*), which are mostly isolated from the vaginal tract (19), are genetically distant from all other *C. albicans* isolates (17), show little genetic diversity (index of nucleotide diversity π for Clade 13 isolates is 0.14 while that for strains of Clades 1–4 and 11 is 0.36), and show distinctive phenotypes, such as the inability to form chlamydospores and to utilize specific carbon sources, notably N-acetylglucosamine, glucosamine, DL-lactate, and trehalose (20). Yet, while showing little genetic diversity, Clade 13 isolates display phenotypic heterogeneity, such as in growth rate, biofilm formation, and filamentation (19). Aside from the uniqueness of Clade 13, there has been limited identification of clade-specific phenotypes (17, 18, 21, 22). Most remarkably, isolates in Clade 1 show a propensity to develop resistance to 5-fluorocytosine (5-FC) due to loss of heterozygosity of a Clade 1-specific heterozygous polymorphism in the *FUR1* gene, which encodes uracil phosphoribosyltransferase (23). Isolates from other clades can become 5-FC resistant through different mutational mechanisms that do not display clade specificity. Isolates from Clades 1 and 3 share another heterozygous polymorphism that affects the *ACE2* gene and whose presence favors hyphal integrity (24). Another example is the observation that Clade 4 isolates appear to share a defect in phosphate metabolism while being indistinguishable from isolates of Clades 1, 2, and 3 for many other phenotypes (25). Yet, as for Clade 13 isolates, isolates from the different clades show diversity in a variety of phenotypes, such as antifungal resistance, morphogenesis, biofilm formation, and virulence (26). The mechanisms that underlie such diversity are generally strain-specific and may result from gain or loss-of-function mutations and regulatory circuit diversification (26).

Exploring the *C. albicans* exometabolome thus requires analyzing a large panel of strains to reveal clade-specific and strain-specific patterns. This is a technical challenge requiring a high-throughput and sensitive method to elucidate differences at a strain level. For such analyses, the use of mass spectrometry (MS) coupled with chromatography has become the most dominant analytical method. However, MS-based techniques are primarily targeted and suffer from three major setbacks when applied to untargeted (profiling) analysis: they are time-consuming as chromatographic separation of the analytes extends analysis time; they can be subjective as they rely on manual curation; and they are limited in their capacity to identify compounds (27, 28). Besides, the effectiveness of MS-based techniques is hampered in samples with a complex matrix (29). For these samples, nuclear magnetic resonance (NMR) is widely employed despite its lower sensitivity, which can result in the lack of detection of low-abundance compounds such as quorum sensing molecules, secondary metabolites, and signaling compounds. Furthermore, for high-throughput applications, metabolic fingerprinting based on metabolic signatures rather than identified metabolites is increasingly preferred (27). In this respect, NMR fingerprinting combined with a statistical analysis was used as a rapid screening test to distinguish between *C. albicans* and *C. dubliniensis*, two closely related species (30). Additionally, using NMR untargeted metabolic profiling of supernatants, discrimination among closely related bacterial species of clinical significance was achieved (31, 32).

Leveraging the advantages of NMR, we used fingerprinting and profiling approaches to investigate exometabolome variation in *in vitro* cultures of 96 clinical *C. albicans* isolates. Our results reveal clade-specific metabolic profiles, particularly Clade 13 isolates being metabolically distinct from the isolates in the rest of the clades. Furthermore, isolate-specific metabolic variations were pervasive, particularly in metabolites involved in central carbon metabolism.

## RESULTS

### A robust experimental strategy for the analysis of *C. albicans* exometabolome

**Fig 1:**
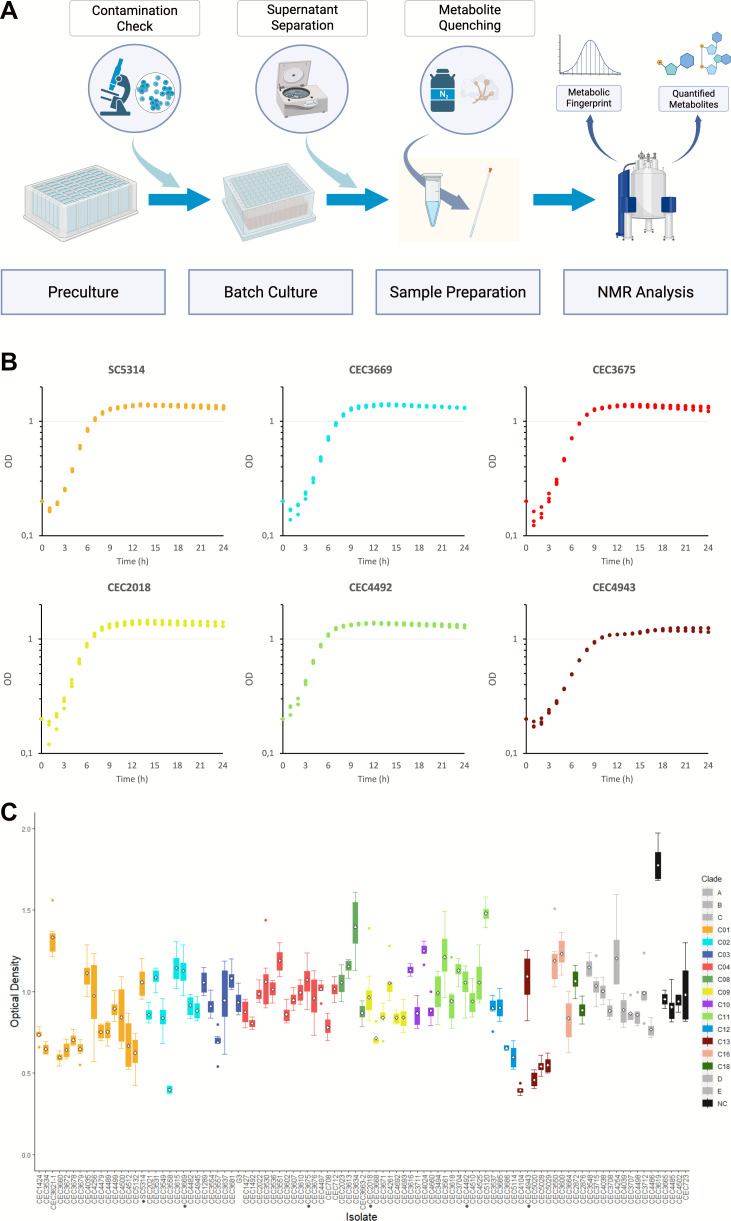
Isolate culture. The culture workflow (**A**) includes duplicate cultures in seven batches. Growth curves of representative isolates (**B**) during a 24-hour period show growth in stationary phase at around 14 h (*n* = 3). Growth parameter values for all isolates are shown in Table S2. Final OD values (**C**) in the batch culture are shown. The representative isolates are labeled with black dots. The data are indicated as mean (white diamond), interquartile range (IQR) (colored boxplots), and whiskers which correspond to 1.5 times IQR (*n* = 14 for representative isolates, except CEC3675 with *n* = 12; *n* = 6 for the rest, except CEC3558 and CEC5120 with *n* = 4).

To enable high-throughput exometabolome analysis of *C. albicans* isolates from different clades, we implemented a 96-well plate-based workflow illustrated in Fig. 1A. Briefly, *C. albicans* isolates were precultured in 48-well plates. The precultures were then checked microscopically for contamination and used to seed 96-well plates, which were incubated for 14 h at 30°C. Supernatants were collected and analyzed by 1H NMR. Then, spectral processing was performed to obtain both the metabolic fingerprint and the quantified metabolite data sets. All strains were cultured, and their supernatants were analyzed in hexaplicate.

A preliminary trial was conducted to assess the feasibility of this approach and to optimize conditions for consistent cell growth and reliable metabolic signals. During this trial, we observed that some isolates displayed low cell densities at the end of the incubation period (Fig. S1). To address this, we increased the starting optical density (OD measured at 600 nm) from 0.1 to 0.2, which enhanced the metabolic signal across isolates. Notably, when cultured under these conditions, all isolates had progressed past the mid-log phase (beyond the inflection point) and were mostly in the early stationary phase at the specified endpoint (14 h) (Fig. 1B; Tables S1 and S2).

Given the large number of samples, we conducted the experiment in batches. For each batch, precultures of around 40 isolates and six reference isolates were grown in 48-well plates overnight. The precultures were then sub-cultured in duplicate in 96-well plates. A control (uninoculated medium) was also included in both precultures and batch cultures. To monitor potential batch effects, we selected six representative isolates from the five most common *C. albicans* clades (Clades 1, 2, 4, 9, and 11) and from Clade 13. These isolates were selected as they showed similar OD at the end of the culture and were included in duplicate in all subsequent batches to test consistency between runs.

In total, we successfully grew 95 out of 96 isolates across seven batches. One isolate (CEC5136 from cluster C) failed to grow. The experiment yielded over 600 samples, as the representative isolates were cultured in duplicate across all batches (*n* = 14), except for CEC3675 (representative isolate from Clade 4), in which duplicate samples in the fifth batch were removed. The remaining isolates were cultured randomly in duplicate across three out of the seven batches (*n* = 6). Additionally, isolates CEC3558 and CEC5120 only grew in two out of three batch cultures (*n* =4).

From the cell culture, we separated and processed the supernatant for 1H NMR analysis, which enabled us to generate the metabolic fingerprint and the quantified metabolite data sets. The metabolic fingerprint data set was obtained by binning (bucketing) the obtained NMR raw spectra, while the quantified metabolite data set was derived through annotation of metabolic features using a database. To generate the quantified metabolite data set, we annotated 40 metabolites and quantified 39 of these using Chenomx NMR Suite (version 8.6). The quantified metabolite data set accounted for around 65% of the peaks covering the spectral region from 0.2 to 10 ppm (Fig. S2).

**Fig 2:**
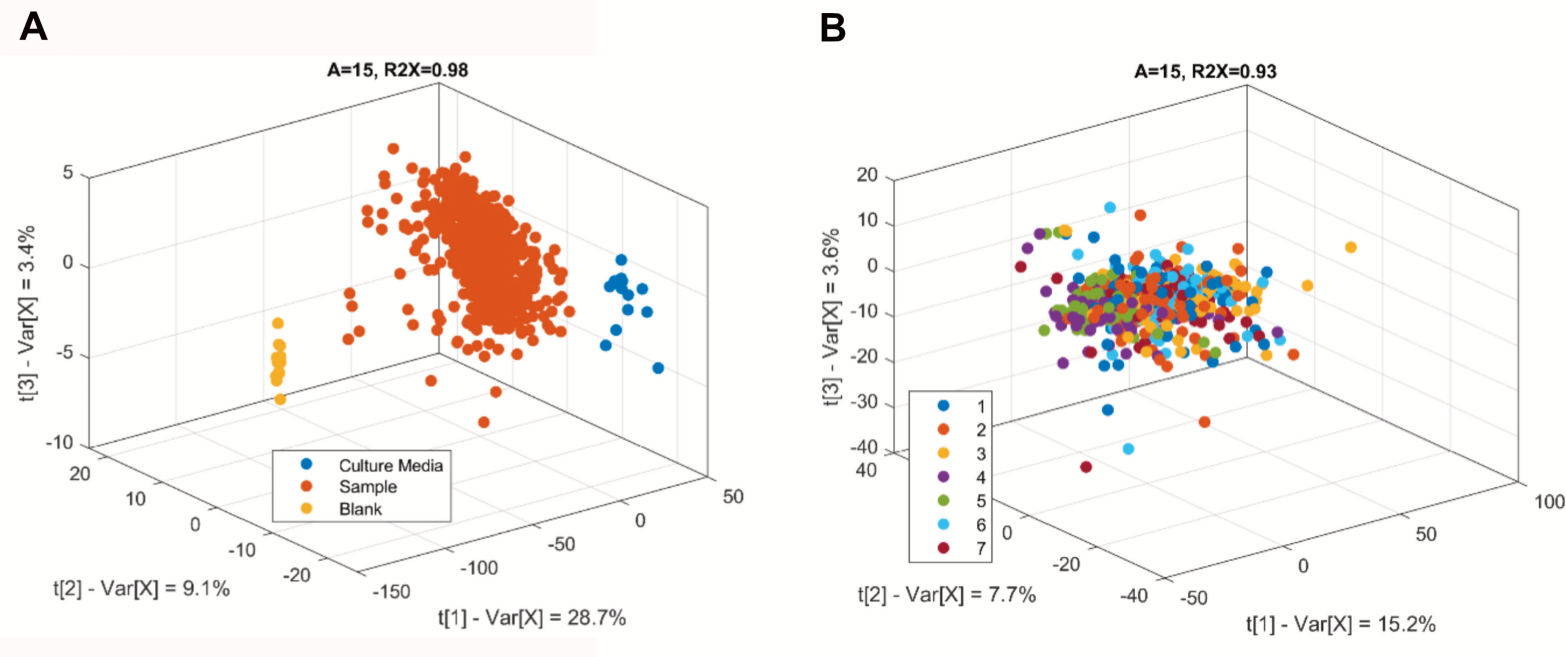
Validation of experimental run data quality using PCA analysis. (**A**) The sample type comparison to determine cross-contamination during culture and sample preparation step. (**B**) Comparison of seven batches to evaluate batch effect. Data points correspond to individual isolate sample replicates, colored by sample type (**A**) and by culture batch (**B**).

We used the metabolic fingerprint data set to assess the quality of the generated data. The unsupervised analysis (principal component analysis, PCA) of this data set showed that the samples were neatly categorized by sample type (Fig. 2A) and that no clustering between batches could be discerned (Fig. 2B). This indicated, first, that there was no cross-contamination during the culture and sample preparation, and second, that variations did not arise from the technical run. To corroborate this analysis, we also used the quantified metabolite data set to assess the metabolite concentrations of the representative isolates in all batches. The assessment showed that all RSD (relative standard deviation) values were below 30%, except for glucose in CEC4943 (Clade 13) (Table S3). Considering that the batch effect was negligible, batch effect correction using the selected representative isolates was not applied.

**Fig 3:**
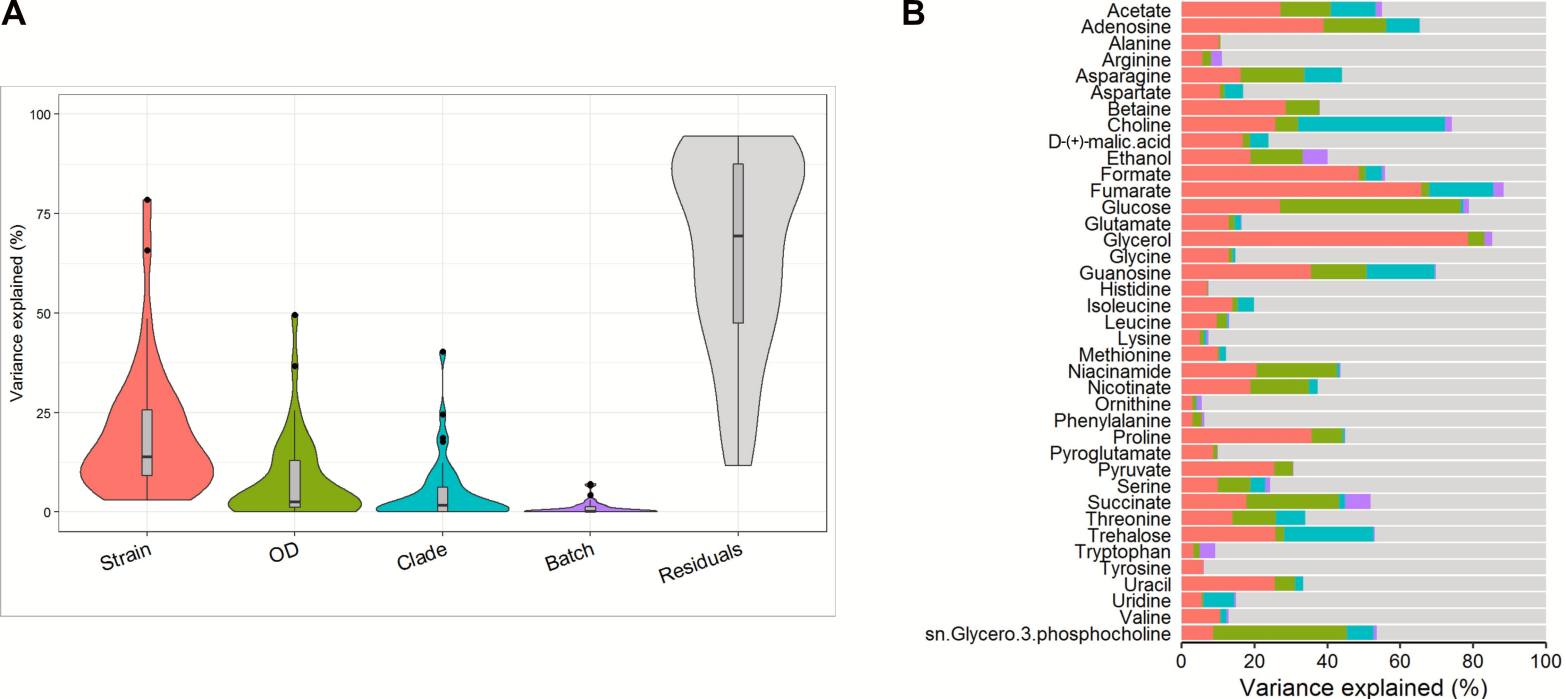
Factors affecting variations. A variance partition analysis (VPA) was performed to determine factors (derived from the metadata and culture-specific parameters) that cause variation among the samples: (**A**) violin plot of the factors that drive the separation and (**B**) variability that can be attributed to these factors for each of the quantified metabolites.

We proceeded with the analysis by combining the quantified metabolite data sets with the metadata provided for each isolate (isolate name, clade affiliation, country of origin, and source—commensal, superficial, invasive, human, animal, and food spoilage) and culture-specific parameters (final OD and batch run). We used this combined data set to perform a variance partition analysis (VPA), which is designed to handle data generated from high-throughput experiments (33). Consistent with the PCA, the VPA also showed that the batch effect was negligible (Fig. 3A). Taking out residual and batch effects, the three remaining sources of variation were all biological factors: strain (isolate), OD (as a proxy for biomass), and clade (Fig. 3A). For other parameters that did not appear prominently in the VPA, such as source and country of origin, our unsupervised analysis (PCA) did not show any clustering (Fig. S3).

During the analysis using the metabolic fingerprint data set, we observed a clustering based on OD. We ran a PCA of this data set and observed an OD gradient, particularly for Clade 13 (Fig. S4). To compensate for the OD effect, we assessed various normalization strategies. Particularly for the quantified metabolite concentrations, we tested ∆ concentration normalization (normalized concentration = [isolate metabolite concentration – control metabolite concentration]/OD) and min-max normalization (normalized value = metabolite concentration/[isolate OD/lowest isolate OD]). However, when we compared the RSD values of the raw concentrations (non-normalized) and the two normalized concentrations, we found that these strategies gave poorer results (Table S4), and the raw concentrations were therefore used for analysis.

### Clade-specific variation

**Fig 4:**
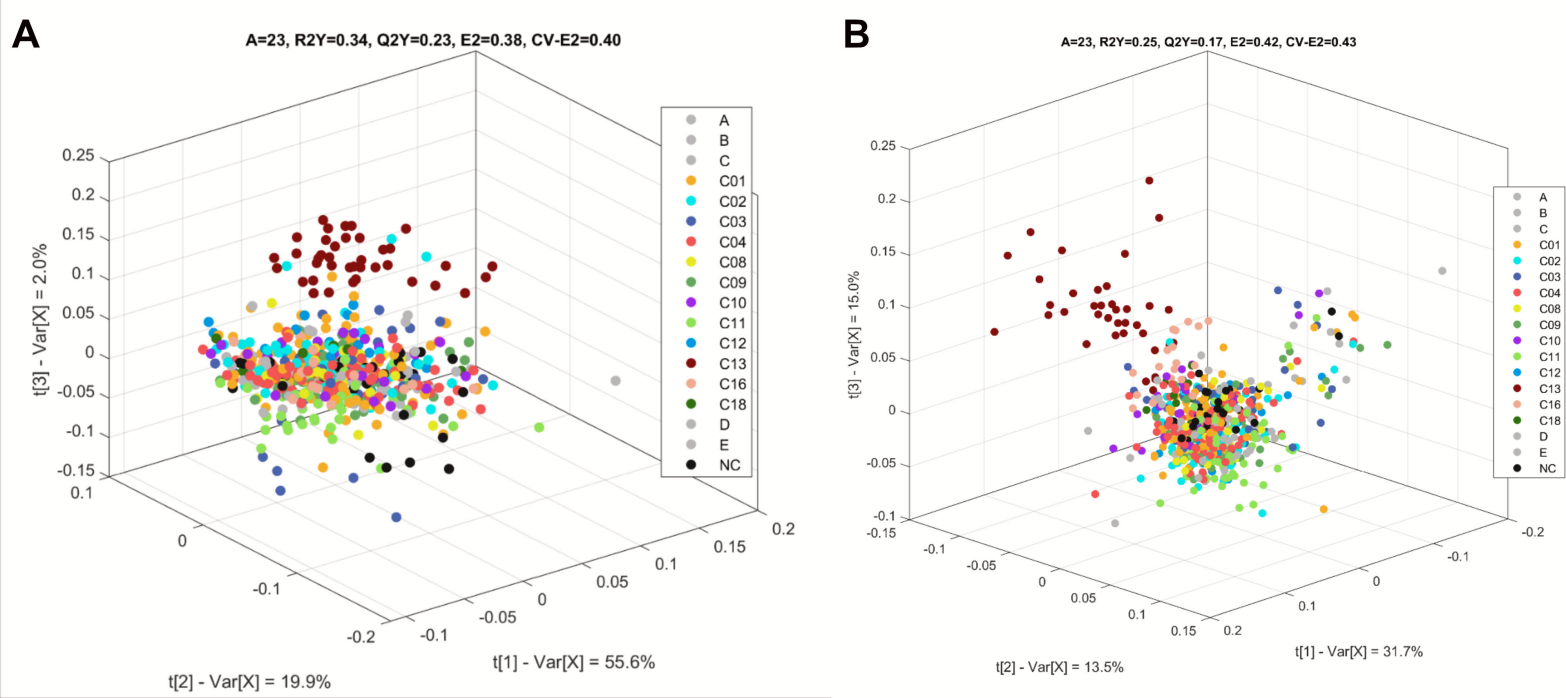
Clade-specific variations. PLS DA of metabolic fingerprint (**A**) and quantified metabolites (**B**) are shown. Data points correspond to individual isolate sample replicates colored by clade.

Using the metabolic fingerprinting and quantified metabolite data sets, we ran a supervised analysis (partial least square discriminant analysis, PLS DA) with clade as the response (grouping) variable (Fig. 4). Even with multiple groups being compared (resulting in the low cumulative explained variance [R2Y] as well as the model predictive accuracy [Q2Y] for metabolic fingerprints [0.34 and 0.23] and quantified metabolite [0.25 and 0.17], respectively), these models distinguished Clade 13 from all other clades. The similarity between both data sets indicated that the quantified metabolite data set captures the high variation within samples, although the reduced R2Y in this data set (Fig. 4B) suggested that the non-quantified features in the metabolic fingerprint data sets (Fig. 4A) also contributed to sample variations. Furthermore, such values were expected as variability attributed to clade affiliation was lower than those ascribed to isolate and OD (Fig. 3).

**Fig 5:**
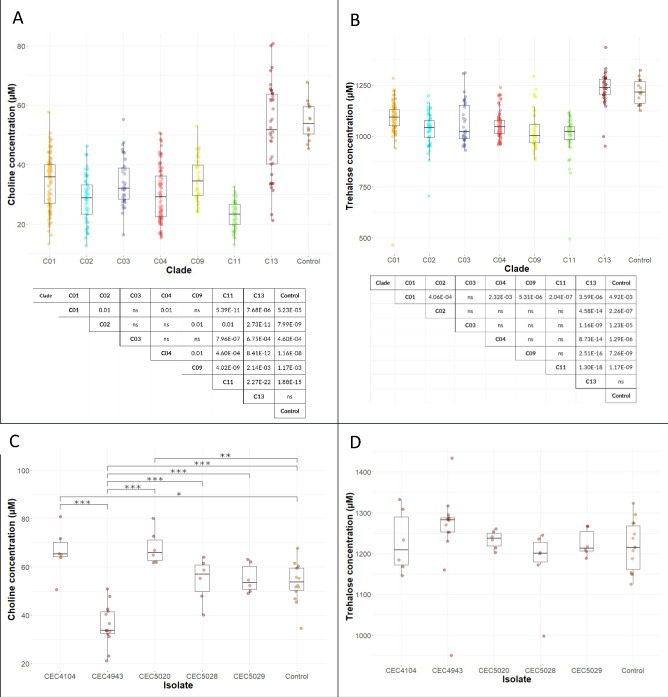
Drivers of clade-specific variation. Clade values for choline (**A**) and trehalose (**B**) concentrations are shown. Both data sets were subjected to Shapiro-Wilk test for normality (*P*< 0.001) prior to Kruskal-Wallis test (A, *P* < 0.001, χ2 = 157.09; B, *P* < 0.001, χ2 = 151.87). The comparison of the seven largest clades and the control is shown (*n* = 14 for representative isolates, except CEC3675 with *n* = 12; *n* = 6 for the rest, except CEC3558 and CEC5120 with *n* = 4). From the data sets, subsets for choline (**C**) and trehalose (**D**) concentrations in Clade 13 were also analyzed. Both subsets were subjected to Shapiro-Wilk test for normality (Clade 13 choline, *P* > 0.05 and trehalose, *P* < 0.001) and choline to Levene’s test for homogeneity of variance (Clade 13 choline, *P* > 0.05) prior to Fisher’s ANOVA (panel C, *P* < 0.001, F = 19.30) and trehalose to Kruskal-Wallis test (panel D, *P* = 0.13, χ2 = 8.57) (*n* =14 for CEC4943 and *n* = 6 for the rest). Post hoc analysis based on pHolm adjusted value was done to separate sample means for (**C**) and sample median for the rest. Significant pairwise comparison is shown in tables in panels A and B and lines with asterisks [* (*P*< 0.05), ** (*P* < 0.01), and *** (*P* < 0.001)] in panels C and D.

To investigate further the possible drivers of clade-specific variation, the VPA was used to determine which metabolites were most responsive to clade affiliation (Fig. 3B). Among the biological variables in the VPA, clade affiliation made the greatest contribution to sample variation in both choline and trehalose. Except for isolates from Clade 13, there were significant clade-wise reduced concentrations (Kruskal-Wallis test, *P*< 0.001) for both choline (Fig. 5A) and trehalose (Fig. 5B) when compared to that of the control (non-inoculated YPD media). Furthermore, these comparisons showed large effect sizes (choline, ε^2^_ordinal_ = 0.36 and trehalose, ε^2^_ordinal_ = 0.35), indicating strong relationship between clade affiliation and metabolite concentration. Examination of metabolite concentrations in isolates belonging to Clade 13 showed that these isolates have unusual choline utilization (Fig. 5C) and did not consume trehalose (Kruskal-Wallis test, *P* = 0.13) (Fig. 5D). Choline concentrations in growth supernatants of Clade 13 isolates displayed unusually wide variation, which varied significantly from that of the control (Fisher’s ANOVA, *P*< 0.001) and registered a large effect size (ε^2^_ordinal_ = 0.64) (Fig. 5D). Two of the isolates showed significantly higher concentrations (CEC4104 and CEC5020, *p_Holm_* adjusted value < 0.001 for both) while one isolate had a significantly lower concentration (CEC4943, *p_Holm_* adjusted value < 0.001).

Since an instance of clade-specific metabolic profile was observed, clade-based analysis was further pursued through supervised analysis (PLS DA). We used both the metabolic fingerprint and quantified metabolites for pairwise clade comparisons, showing that clades were distinct from each other in their metabolic profiles (Fig. S5). These pairwise comparisons showed high cumulative explained variance (R2Y values higher than 0.82 for metabolic fingerprints and 0.59 for quantified metabolites) and high model predictive accuracy (Q2Y values higher than 0.52 for metabolic fingerprints and 0.44 for quantified metabolites). Furthermore, consistent with the VPA, all pairwise comparisons involving Clade 13 showed trehalose and choline as having the highest VIP (variable importance in the projection) scores. This suggested that these two metabolites are the top discriminants of the variations when Clade 13 is compared with other clades.

### Isolate-specific variation

**Fig 6:**
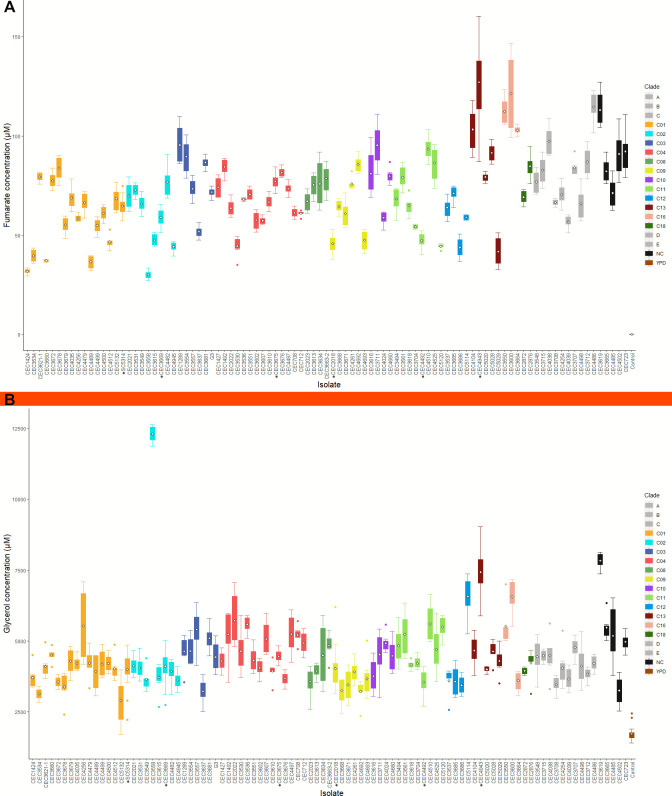
Isolate-specific variation. Variation was most pronounced in (**A**) fumarate and (**B**) glycerol. Isolates with a black dot are representative isolates. Data are indicated as mean (white diamond), interquartile range (IQR) (colored boxplots), and whiskers which correspond to 1.5 times IQR (*n* = 14 for representative isolates, except CEC3675 with *n* = 12; *n* = 6 for the rest, except CEC3558 and CEC5120 with *n* = 4).

The most prominent biological factor driving variation in the VPA could be attributed to factors inherent to the isolates. When the effect of this inherent isolate variability was assessed on individual metabolites, it was observed that fumarate and glycerol were the most responsive (Fig. 3B), showing increased concentrations when compared with the control. These increases were most visible for fumarate, which ranged from 30 to 127 µM but was detected only at negligible levels in the control medium. A striking feature of the quantified metabolite data set was the pronounced increase in the glycerol production of Clade 2 isolate CEC3558 (12,296 µM), which is seven times the level observed in control and almost three times more than the mean concentration observed for other isolates within Clade 2 (Fig. 6B). This isolate survived only in two out of three duplicate cultures and showed poor growth (OD_600_ = 0.4 + .03 (mean ± SD), *n* = 4 replicates) compared with the rest of the isolates (Fig. 1).

However, while both metabolites showed wide concentration differences between isolates (Fig. 6), it was notable that there were low variations observed within replicates for each isolate. The RSD is below 20% for fumarate and 30% for glycerol, indicating that culture conditions did not vary much between batch runs. Given this, we ascertained the high isolate-specific variations observed in the VPA by performing within-clade comparison for these two metabolites (Table S5). For both metabolites, differences in isolate concentrations were significant in all comparisons (*P* < 0.001 in all comparisons, except for glycerol in Clade 9, *P* = 0.04). The computed effect sizes were also large for both fumarate (ε^2^_ordinal_ ranging from 0.87 to 0.95) and glycerol (ε^2^_ordinal_ ranging from 0.27 to 0.78), indicating that metabolite concentration was strongly related to differences in isolate.

Considering that metabolite production can be a function of biomass, we evaluated if culture OD affected metabolite concentration. We performed correlation analysis on both fumarate and glycerol (Fig. S6). Although highly significant, the positive correlation between OD and fumarate (Spearman rank correlation, *P* < 0.001 and ρ = 0.18) and glycerol (*P* < 0.001 and ρ = 0.22) concentrations, respectively, was very weak. Consistent with the VPA, these analyses indicated that while OD affected the concentrations of both metabolites, OD contribution was largely negligible.

**Fig 7:**
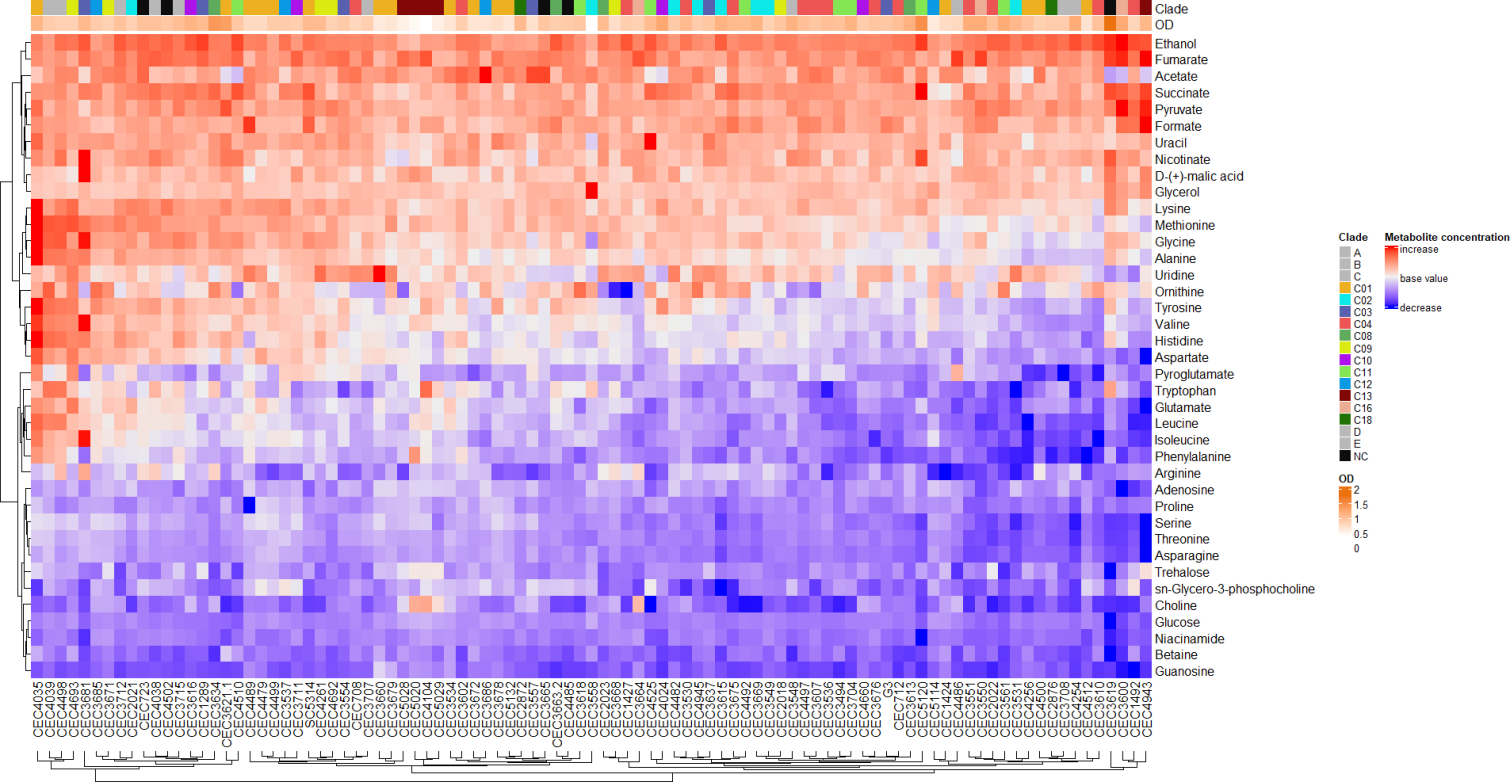
Heat map of relative metabolite utilization. Color intensities represent utilization with shades of blue indicating a decrease (consumption) and red indicating an increase (production) of metabolites relative to control values.

Since we also observed isolate-specific variation in metabolite concentrations, we constructed a heatmap using the quantified metabolite data set to determine the relative metabolite utilization of each isolate (Fig. 7). The heatmap showed a general reduction in metabolites used as precursor molecules for central carbon metabolism. Consequently, there was an increase of metabolites produced as by-products of glycolysis and as intermediates of the TCA (tricarboxylic acid)/Krebs cycle (Table S6). However, except for the close proximity of four out of five Clade 13 isolates, these utilization patterns did not correspond to clade nor OD. This strongly suggested that metabolite utilization was isolate-specific.

## DISCUSSION

In this study, we compared the exometabolomes of a collection of *C. albicans* isolates spanning a wide genomic diversity. Notably, most of the tested isolates have not been exposed to antifungals previously, reducing the possible phenotypic effects of mutations, such as genome instability and genome rearrangements, that can be triggered by exposure to antifungals such as azoles and echinocandins (34). We also selected a sampling time at which the isolates had uniformly reached early stationary phase. Terminating cultures at a predetermined time point allowed synchronization of the culture and analytical workflow by processing all samples at once, enabling full randomization. However, we propose that this workflow could be improved using nutrient-limited media, as this would allow the observation of clearer metabolic differences. Although the use of YPD was found suitable for *in vitro* investigations involving both metabolomic and lipidomic analyses (35), the use of a rich medium provides excess nutrients that over-fuel cellular processes, masking subtle differences that might have been inherent to a clade or a particular isolate. Since our workflow relies on the identification of metabolic changes from NMR data, the use of limited media in a miniaturized workflow should be optimized such that variations in the metabolic signals could be detected.

Having confidence that we had implemented a robust experimental workflow for exometabolome characterization, we proceeded to evaluate the drivers of exometabolome variations in *C. albicans*. Our analysis of the metadata variables indicated that the source of isolates (commensal, superficial, invasive, human, animal, and food spoilage) did not underlie any metabolic variability. While this may in part reflect the limitation of NMR (see below), it is consistent with previous findings (18) and is expected, as phenotypic responses are less pronounced and less diverse in *in vitro* cultures than under *in vivo* conditions (3, 36). The complexities of the human host force *C. albicans* to respond and to adapt, as evidenced by a recent report in which metabolic signatures of *C. albicans* isolated from the blood, respiratory tract, and vaginal niche were compared through 1H NMR and were shown to differ (37). It ensues that exometabolome profiling of *in vitro*-cultivated isolates, as performed in our study, cannot be used as predictor of the severity of the infection these isolates could cause in a patient. Unlike previous observations that noted geographic enrichment of the clades (18, 22), we did not see clustering based on country origin.

Our results point to the uniqueness of the isolates’ metabolic profiles as being the most predominant biological factor causing variations. We observed that isolate-specific variability occurs within clades and is centered on metabolites linked to central carbon metabolism. Wang and colleagues (38) have shown wide inter-isolate variations in *C. albicans* gene expression, particularly in genes involved in cell cycle regulation, lipid metabolism, and carbohydrate metabolism. In aerobic conditions and in the presence of abundant carbon sources, yeast cells use inefficient metabolic routes and convert carbon sources into typical end products, such as ethanol, acetate, and lactate (39). Excluding lactate, which we have not quantified, we observed increases in both ethanol and acetate production. Furthermore, upon reaching capacity of the intracellular compartment due to elevated rates of glycolysis, secretion of metabolites is observed, a phenomenon referred to as overflow metabolism (40). As such, the increases in extracellular concentrations of glycolysis by-products and metabolites involved in the TCA cycle are expected. The two metabolites showing highest isolate-specific variation were also involved in central carbon metabolism—glycerol is a product of the shunt of glycolysis, while fumarate is an intermediate metabolite in the TCA cycle (39).

Regarding isolate-specific variations, the most striking observation is the high production of glycerol by strain CEC3558, a slow-growing commensal isolate. Glycerol is produced and accumulated in cultures and conditions due to settling (biofilm formation), osmotic stress, or citric acid stress—a response mediated via the Hog1 MAPK (high osmolarity glycerol 1 mitogen-activated protein kinase) pathway (41–43). One plausible reason could be that strain CEC3558 is still producing, or has not yet consumed, glycerol, considering that *C. albicans* produces glycerol in the early phase of growth and begins consumption only when preferred carbon sources are depleted (41). The shift from net glycerol production to assimilation is technically challenging to capture in high-throughput culture workflows, but could be further investigated in a dedicated study involving flux analysis using isotope labeling. What is clear is that this peculiar characteristic of strain CEC3558 could only be unraveled through a comparison of a large number of isolates with wide genetic diversity.

Clade-specific variation also figured prominently as the cause of biological variation. The observed between-clade variation (isolates within a clade share metabolic characteristics that are distinguishable from isolates in another clade) was dominated by the distinctiveness of the Clade 13 (*C. africana* isolates) exometabolome, which is phylogenetically distinct due to lower heterozygosity possibly brought about by an ancient genome-wide LOH (loss of heterozygosity) event (17). Among many carbon sources, it lacks the ability to assimilate extracellular trehalose (19), a finding further confirmed by this study. While the inability to assimilate trehalose has been empirically observed (44), the mechanisms involved are likely not fully understood. In *Saccharomyces cerevisiae,* utilization of exogenous trehalose is mediated by the Ath1 extracellular acid trehalase (45). Pedreno et al. (46) found a cell-wall-linked enzyme (Atc1), encoded by the *ATC1* gene (an ortholog of the *S. cerevisiae ATH1* gene), in *C. albicans* that similarly mediates the utilization of trehalose from external sources. While it is possible that Clade 13 isolates could have a defect in *ATC1* expression or Atc1 function, mining the genomes of the 96 *C. albicans* isolates did not reveal obvious mutations in *ATC1* that could explain the trehalose utilization defect of Clade 13 isolates.

Clade 13 isolates also showed, for the first time, an unusual utilization of choline—another metabolite that showed greatest response to clade affiliation. Exogenous choline and ethanolamine are metabolized by *C. albicans* via the Kennedy pathway into either phosphatidylethanolamine (PE) or phosphatidylcholine (PC), phospholipids that constitute major components of the eukaryotic membrane (47). This pathway proceeds through a three-step enzymatic process, with the last enzyme being Ept1 in *C. albicans* (47, 48). Overexpression of the *EPT1* gene leads to increased PE synthesis, causing hypervirulence in a murine model of systemic infection (48). Hypervirulence is a consequence of post-transcriptional changes and is associated with increased chitin content and increased hyphal length (49). However, it has been shown that uptake of exogenous ethanolamine is not sufficient to induce virulence when not supported by PE *de novo* synthesis from cytidine diphosphate-diacylglycerol (CDP DAG) (50), indicating that exogenous choline plays a more dominant role than exogenous ethanolamine in virulence. Thus, the unusual choline utilization of Clade 13 isolates could be due to a defect in the Ept1-mediated enzymatic step of the Kennedy pathway. The muted pathogenic characteristics of these isolates are consistent with this hypothesis. Again, mining the genomes of the 96 *C. albicans* isolates did not reveal obvious mutations in *EPT1* that could explain the choline utilization defect of Clade 13 isolates.

While trehalose and choline were obvious drivers of clade-specific metabolic differences, variations due to other metabolites most likely exist and contribute, to a certain extent, to the observation that isolates belonging to the same genetic clade group together based on metabolic properties. Furthermore, while these metabolites represent important coverage of the metabolome, as reflected in the similarity between the metabolic fingerprints and quantified metabolites PLS DA plots, they comprise only a fraction of the metabolic features present in the sample. Indeed, the pairwise comparison of the metabolic fingerprints shows higher R2Y and Q2Y values than those of the quantified metabolite data sets. While clade-specific variations were dominated by the distinct Clade 13 metabolic profile, the pairwise clade comparison showed that, globally, isolates within a clade share collective metabolic signatures distinguishable from those of isolates from another clade. In general, metabolic complexity mostly emerges from genetic processes and their consequences on intracellular metabolism (51). Obtaining each of the isolate’s intracellular metabolic profiles and linking these with genomic data would be an effective strategy for determining the processes enriched or unique to each clade. Yet, this is a challenging endeavor, especially for diploid species with predominantly clonal reproduction and when only a limited number of isolates have been characterized for their exometabolome. We have so far not succeeded in linking exometabolome profiles with genetic differences, as exemplified in the case of trehalose and choline utilization defects of Clade 13 isolates.

Through a robust high-throughput strategy, we have shown that *C. albicans* isolates have divergent metabolic profiles that can be observed within clade (isolate-specific) and between clades (clade-specific). While between-clade variation was dominated by the distinctiveness of Clade 13 metabolic profiles, we have also demonstrated that all clades had distinct exometabolome profiles. The distinction of Clade 13 runs parallels with the recent genomic classification (17). Our findings confirmed the role of trehalose and, at the same time, uncovered the possible role of choline as drivers of the separation of Clade 13 isolates from the other isolates based on their exometabolome properties. Furthermore, to our knowledge, our work generated some of the most extensive data on the *C. albicans* metabolic profile. These data sets provide useful resources for the scientific community to further explore.

With these, and in consonance with the previous finding that *C. albicans* exists as a consortium of isolates with a wide genomic background in the human host (36), we suggest that work involving metabolic profile should consider *C. albicans* as a complex of unique isolates and not as a singular entity. As this would entail screens involving a large number of isolates, the high-throughput workflow implemented here will be useful in carrying out these investigations. Yet, despite the robustness of the NMR-based workflow used in this study, intrinsic limitations of NMR spectroscopy should be acknowledged. In particular, the comparatively low sensitivity of NMR restricts detection to abundant metabolites, primarily reflecting variations in primary metabolism, while low-abundance compounds, such as secondary metabolites, quorum sensing molecules, signaling compounds, and volatile metabolites, are likely not detected. These limitations may have contributed to the absence of detectable exometabolomic differences associated with the clinical source of the isolates under the *in vitro* conditions used. Accordingly, the metabolic signatures identified here are not directly predictive of infection severity or clinical outcome but rather provide a robust framework for investigating strain- and clade-level metabolic diversity. Complementary mass spectrometry–based approaches and host-mimicking conditions will be required to extend these findings toward clinically relevant traits.

## MATERIALS AND METHODS

### Clinical isolates

The 96 clinical *C. albicans* isolates used in this study (Table S1) were from the collection of the Fungal Biology and Pathogenicity Unit of the Institut Pasteur (Paris, France). These genome-sequenced isolates are representative of *C. albicans* genetic diversity, belonging to 12 out of 18 established MLST clades (80 isolates), while the rest either formed smaller genomic clusters (clusters A–E) (11 isolates), or are non-clustering (NC) isolates (singletons) (5 isolates). The assignment of these isolates to genomic clades was performed using multi-locus sequence typing (MLST) and confirmed by genome sequencing (17). All isolates are euploid. Prior to the experiment, the isolates were re-cultured in YPD, preserved in 25% glycerol, and stored at −80°C.

### Culture

Precultures were initiated by activating the cryopreserved isolates in a 48-deepwell culture plate with 2.5 mL YPD. The plate was covered with sterile plate filter (Breathseal gas permeable filter, Greiner Bio-One) and placed in a shaking incubator (INCU line ILS 6, VWR) at 30°C and 250 rpm for 24 h. After incubation, culture OD was determined using a spectrophotometer (Ultra Spec 10 Cell Density Meter, Amersham Biosciences). Then, the final cultures were initiated by infusing fresh YPD to the cell suspension at an initial concentration of OD = 0.2 and run for 14 h in preculture conditions (30°C and 250 rpm). Both the precultures and final cultures included non-inoculated YPD media as controls.

After 14 h of incubation, culture OD was determined using a microplate reader (Infinite M200 Pro, Tecan). Then, 300 µL of supernatant was obtained through centrifugation (1,500 × *g*, 10 min, 10°C). The obtained supernatant was transferred to a new plate, which was subsequently covered with aluminum seal and quenched in liquid nitrogen to arrest metabolic activity. The plate was then stored at −80°C prior to analysis.

In between the precultures and final cultures, the cultures were checked for microbiological contamination. From the precultures, 1 mL cell suspension was taken. The supernatant from this aliquot was removed, and the cells were washed with PBS (Dulbecco PBS, Thermo 14190169). The washed cells were then fixed in 500 µL pure ethanol. From the fixed cells, 10 µL were obtained and Gram-stained using a commercial kit (Sigma 77730-1KT-F). The Gram-stained cells were fixed in a glass slide (Sigma Eukitt 03989-100ML) before being visualized in brightfield at 40× magnification (Nanozoomer S60, Hamamatsu). Images were then individually checked using the NDP.view2 software (Hamamatsu).

The initial OD = 0.2 was based on a trial culture using the workflow. The trial culture of all isolates (*n* = 2) at initial OD = 0.1 resulted in low final culture OD (Fig. S1), thus the increase in the initial OD. Furthermore, since the experiment was designed to be run in batches, the trial culture was used to select six isolates with similar final OD values. These representative isolates (SC5314, CEC2018, CEC3669, CEC3675, CEC4492, and CEC4943) were used to assess if there is variability between batches.

On the other hand, the 14-hour final culture period was based on the growth curves generated through a 24-hour period. For the growth curve generation, the isolates were cultured based on the preculture conditions. Then, for each isolate, triplicate preparations of 100 µL cell suspension at initial OD = 0.2 were transferred to a 96-well flat-bottom plate. The plate was covered with sterile adhesive film (Dutscher Breathe-Easy or Breathe-Easier), and then OD readings were taken every hour for 24 h using LogPhase600 (Agilent) following the manufacturer’s recommendation (30°C and 800 rpm).

In total, seven culture batches were done. The representative isolates were cultured in duplicate in all batches (*n* = 14 replicates), while the rest of the isolates were cultured in duplicate in three out of seven batches (*n* = 6 replicates). In each batch culture, the isolate grouping and the position of the isolate in the culture plates were randomized.

### Extracellular metabolite sample preparation

The quenched supernatants were thawed in a thermoshaker (1,000 rpm, 1.5 h, 10°C). To remove cell debris, the supernatants were filtered through a 0.2 µm filter (Acroprep Advance 96 well 350 µL Filter Plate, Pall Corporation) using centrifugation (1,500 × *g*, 10 min, 10°C). From the collected supernatant, the proteins were removed via centrifugation (1,500 × *g*, 1 h, 10°C) through a 10 kDa cut-off filter (Acroprep Advance 96 well 350 µL Filter Plate, Pall Corporation). Prior to use, the 0.2 µm filter was prewashed in 200 µL Milli Q water, and the 10 kDa cut-off filter was prewashed in 200 µL Milli Q water containing 0.05 N NaOH to remove contaminants.

Aliquots of the filtered supernatants (135 µL) were then transferred to a round-bottom 96-well plate and mixed with 45 µL of buffered internal standard (DSS sodium trimethylsilylpropanesulfonate + DFMTP difluorotrimethylsilanylphosphonic acid). Similarly, succinate standard (135 µL) at a defined concentration was mixed with the buffered internal standard (45 µL). The succinate standard mixture was prepared in triplicates. From the 180 µL mixture, 155 µL was transferred to a 3 mm NMR tube, and the tube cap was plugged with a bead. Then, the tubes were centrifuged gently (200 × *g*, 1 min) and stored at 4°C prior to analysis. The succinate standard mixture was run with the samples and used to determine the DSS concentration.

### NMR spectrum acquisition and preprocessing

The prepared supernatants were analyzed using one-dimensional proton spectrum acquired at 298 K on a 600 MHz NMR (Ascend NMR Avance III HD, Bruker). The NMR was equipped with a quadruple cryogenic inverse probe for 1H/13C/15N/31P detection. The 1D proton spectra were acquired using the *noesygppr1d* acquisition sequence with TopSpin software (version 4.0.8, Bruker). The spectra were recorded during 3.89 s acquisition time into 64 k data points and 14 ppm spectral width. Solvent signal suppression was achieved at a mixing time of 70 ms and water signal presaturation during a 4 s relaxation delay. Each spectrum was collected in 32 scans, for a total time of 4.47 min. The spectra were processed using 0.3 Hz line broadening prior to Fourier transformation. Then, spectral alignment was done through phase adjustment, baseline correction, and axis calibration to reposition the DSS peak at 0 ppm.

### Metabolic fingerprint feature extraction

Fingerprint features were obtained from the NMR raw spectra using the Amix software (version 4.0.1, Bruker). Before processing, the water region (4.52–4.68 ppm) was deleted, and the right and left borders were set at 0.02 and 10 ppm, respectively. Then, spectral bucketing was fixed at 0.0005 ppm size. Finally, the non-normalized fingerprint features were converted into readable format (.csv data matrix).

From these fingerprint features, extraction of NMR spectral data was performed using an in-house fingerprinting workflow (Biotracs) based on MATLAB (version R2019a). This workflow consisted of spectral alignment using the icoshift toolbox (version 1.3.2) (52) and feature grouping using covariance analysis. After feature grouping, a filter was applied to only keep NMR features having intensity greater than a predefined threshold of 10^5^. This threshold corresponds to the level of the background noise in the spectra. The final data set was then used for statistical analysis.

### Metabolite annotation and quantification

From the raw NMR spectra, spectral peaks were annotated and subsequently quantified using the Chenomx NMR Suite (version 8.6). The DSS concentration, obtained through integration with succinate standard solution, was used for CSI (chemical shift indicator) correction in the Chenomx Profiler. Metabolite annotation and quantification were done based on HMDB (Human Metabolome Database) (Table S7). Chenomx DiscoverM was used to export metabolite raw values into readable data matrix.

### Data analysis

The metabolic fingerprint data set was used to assess clustering based on sample type, batch run, source, and country of origin. An unsupervised analysis (PCA) was run on the data set using a reduced and centered normalization. On the other hand, both the metabolic fingerprint and quantified metabolite data sets were used for clade pairwise comparison. The pairwise comparison was done independently for each data set using the supervised analysis (PLS DA). Furthermore, the quantified metabolite data set was used to run the VPA. The VPA, which summarizes the contribution of technical and biological variations in terms of the fraction of variation explained (FVE), was run using a linear mixed model framework (33). This data set was also used to construct a heatmap. In constructing the heat map, metabolite concentrations in the control (non-inoculated YPD media) were subtracted from those of the cell culture. The isolate values were then scaled to the control values by dividing the highest (for positive values) and the lowest (for negative values) concentration by its absolute value. Thus, values higher than zero indicate increase (production), while those that are lower than zero indicate decrease (consumption) in metabolite concentrations.

For metabolites that drive clade-specific properties based on the VPA (choline and trehalose), a between-clade comparison was run using either parametric (one-way ANOVA) or non-parametric (Kruskal-Wallis test) analysis. The same analyses were done for within-clade comparison of choline and trehalose in Clade 13, as well as for metabolites showing the highest isolate-specific variation (fumarate and glycerol). The type of analysis for each metabolite was based on the test for normality (Shapiro-Wilk) and equality of variance (Levene’s test) for normally distributed data. Furthermore, correlation analysis between OD and the metabolites fumarate and glycerol was done using Spearman rank correlation, following the Shapiro-Wilk test for normality. Effect size was also computed for all comparisons and interpreted based on Cohen (53). All analyses were performed at α = 0.05.

The PCA, PLS DA, and the VPA were run through the Biotracs in MATLAB (version 2019a). The R (4.2.1) software was used to compute for growth parameters (Growthcurver) (54), generate the heat map (Complexheatmap) (55), and analyze the data (ggstatsplot) (56).

## Data Availability

The data files are available at the Metabolomics Workbench under study ID ST002392 (https://www.metabolomicsworkbench.org/data/DRCCMetadata.php?Mode=Study&StudyType=&StudyID=ST002392).

## SUPPLEMENTAL MATERIAL

Supplemental material aem.01512-25-s0001.docx Tables S1 to S7; Fig. S1 to S6.

ASM does not own the copyrights to Supplemental Material that may be linked to, or accessed through, an article. The authors have granted ASM a non-exclusive, world-wide license to publish the Supplemental Material files. Please contact the corresponding author directly for reuse.
