# Supplementary material for: Exometabolome variation in the fungal pathogen of humans Candida albicans reveals specificities at the genetic clade and strain levels
Source: Appl Environ Microbiol. 2026 Mar 18;92(4):e01512-25. doi: 10.1128/aem.01512-25 (PMC13101467; doi:10.1128/aem.01512-25)
Supplement: Supplemental material — Tables S1 to S7; Fig. S1 to S6. [file aem.01512-25-s0001.docx]

**Exometabolome variation in the fungal pathogen of humans *Candida albicans* reveals specificities at the genetic clade and strain levels**

**Supplemental material**

**Supplemental Table 1: Information for 96 *C. albicans* isolates.** Entries in bold face are representative isolates. Mean+SD values are shown for Culture_OD (n = 14 for representative isolates, except CEC3675 with n = 12 replicates and n = 6 for the rest except CEC3558 and CEC5120 with n = 4).

| Isolate | CLADE | Culture OD | OTHER_NAME | Country | Source | First described in |
| --- | --- | --- | --- | --- | --- | --- |
| CEC3548 | A | 1.15+0.06 | C80 | Belgium | commensal | Bougnoux et al., 2006 |
| CEC3715 | A | 1.03+0.11 | SATURRM6 | France | superficial | Bougnoux et al., 2008 |
| CEC4038 | A | 1.00+0.06 | 321 | UK | superficial | Ropars et al., 2018 |
| CEC3708 | B | 0.88+0.05 | EGPURRM17 | France | superficial | Bougnoux et al., 2008 |
| CEC4254 | B | 1.21+0.23 | CHV1 | France | Invasive | Garnaud et al., 2015 |
| CEC4039 | C | 0.89+0.09 | 322 | UK | superficial | Ropars et al., 2018 |
| CEC5136 | C | - | UMIP 1663.86 | UK | - | - |
| CEC3707 | D | 0.87+0.06 | EGPURRM15 | France | superficial | Bougnoux et al., 2008 |
| CEC4498 | D | 0.86+0.08 | - | unknown | food spoilage | Ropars et al., 2018 |
| CEC3712 | E | 0.99+0.14 | SATURRM2 | France | superficial | Bougnoux et al., 2008 |
| CEC4486 | E | 0.77+0.05 | - | unknown | food spoilage | Ropars et al., 2018 |
| CEC3619 | NC | 1.78+0.13 | C87 | Belgium | commensal | Bougnoux et al., 2006 |
| CEC3665 | NC | 0.95+0.04 | LRBURRM2 | France | superficial | Bougnoux et al., 2008 |
| CEC4485 | NC | 0.91+0.10 | - | unknown | food spoilage | Ropars et al., 2018 |
| CEC4502 | NC | 0.94+0.04 | BEYM05 | France | superficial | Sitterlé et al., 2020 |
| CEC723 | NC | 1.06+0.23 | HAM | France | superficial | Ropars et al., 2018 |
| CEC1424 | 1 | 0.73+0.04 | Niger12 | Niger | superficial | Odds et al., 2007 |
| CEC3534 | 1 | 0.65+0.03 | M20 | Morocco | commensal | Sdoudi et al., 2014 |
| CEC3621-1 | 1 | 1.33+0.13 | - | Brazil | commensal | Ropars et al., 2018 |
| CEC3660 | 1 | 0.60+0.03 | ABCURRM3 | France | superficial | Bougnoux et al., 2008 |
| CEC3672 | 1 | 0.64+0.05 | TNMHCRC4 | France | Invasive | Bougnoux et al., 2008 |
| CEC3678 | 1 | 0.70+0.04 | SLSURRC14 | France | superficial | Bougnoux et al., 2008 |
| CEC3679 | 1 | 0.65+0.05 | SLSURRC15 | France | superficial | Bougnoux et al., 2008 |
| CEC4035 | 1 | 1.11+0.11 | 316 | UK | superficial | Ropars et al., 2018 |
| CEC4256 | 1 | 0.97+0.26 | FON1 | France | superficial | Garnaud et al., 2015 |
| CEC4479 | 1 | 0.75+0.05 | - | unknown | food spoilage | Ropars et al., 2018 |
| CEC4489 | 1 | 0.75+0.06 | - | unknown | food spoilage | Ropars et al., 2018 |
| CEC4499 | 1 | 0.90+0.07 | - | unknown | food spoilage | Ropars et al., 2018 |
| CEC4500 | 1 | 0.84+0.20 | - | unknown | food spoilage | Ropars et al., 2018 |
| CEC4512 | 1 | 0.67+0.16 | BOIN30 | France | superficial | Sitterlé et al., 2020 |
| CEC5132 | 1 | 0.62+0.11 | UMIP 1332.82 / ATCC 26278 | unknown | superficial | - |
| **SC5314** | **1** | **1.06+0.09** | **SC5314** | **USA** | **invasive** | **Gillum et al., 1984** |
| CEC2021 | 2 | 0.86+0.05 | APRHRM2 | France | invasive | Bougnoux et al., 2008 |
| CEC3531 | 2 | 1.09+0.06 | D1_X | France | commensal | Schönherr et al., 2017 |
| CEC3549 | 2 | 0.84+0.09 | C82 | Belgium | commensal | Bougnoux et al., 2006 |
| CEC3558 | 2 | 0.40+0.03 | C39 | Belgium | commensal | Bougnoux et al., 2006 |
| CEC3615 | 2 | 1.15+0.11 | M46 | Morocco | commensal | Sdoudi et al., 2014 |
| **CEC3669** | **2** | **1.13+0.09** | **TNMURR22** | **France** | **superficial** | **Bougnoux et al., 2008** |
| CEC4482 | 2 | 0.92+0.07 | - | unknown | food spoilage | Ropars et al., 2018 |
| CEC4945 | 2 | 0.88+0.06 | VPCI 84/P/13 | India | superficial | Sharma et al., 2014 |
| CEC1289 | 3 | 1.06+0.07 | Bougn10 | France | invasive | Bougnoux et al., 2002 |
| CEC3554 | 3 | 0.91+0.06 | BCHUR6 | France | superficial | Bougnoux et al., 2008 |
| CEC3557 | 3 | 0.69+0.08 | C11 | Belgium | commensal | Bougnoux et al., 2006 |
| CEC3637 | 3 | 0.95+0.22 | Niger14 | Niger | superficial | Odds et al., 2007 |
| CEC3681 | 3 | 1.08+0.07 | EGPHCRC4 | France | invasive | Bougnoux et al., 2008 |
| CEC5255 | 3 | 0.93+0.08 | G3 | France | commensal | Sitterlé et al., 2020 |
| CEC1427 | 4 | 0.87+0.07 | Bougn08 | France | invasive | Bougnoux et al., 2002 |
| CEC1492 | 4 | 0.80+0.03 | - | unknown | unknown | Odds et al., 2007 |
| CEC2022 | 4 | 0.99+0.05 | GEFrouen | France | invasive | Schönherr et al., 2017 |
| CEC3530 | 4 | 1.06+0.21 | C1_X | France | commensal | Schönherr et al., 2017 |
| CEC3536 | 4 | 1.01+0.06 | M23 | Morocco | commensal | Sdoudi et al., 2014 |
| CEC3551 | 4 | 1.19+0.08 | EGPURM24 | France | superficial | Bougnoux et al., 2008 |
| CEC3602 | 4 | 0.86+0.06 | M52 | Morocco | commensal | Sdoudi et al., 2014 |
| CEC3607 | 4 | 0.95+0.05 | M3 | Morocco | commensal | Sdoudi et al., 2014 |
| CEC3610 | 4 | 0.99+0.06 | M36 | Morocco | commensal | Sdoudi et al., 2014 |
| **CEC3675** | **4** | **1.06+0.11** | **SLSHCRC2** | **France** | **invasive** | **Bougnoux et al., 2008** |
| CEC3676 | 4 | 0.96+0.15 | SLSURRC1 | France | superficial | Bougnoux et al., 2008 |
| CEC4497 | 4 | 1.02+0.05 | - | unknown | food spoilage | Ropars et al., 2018 |
| CEC708 | 4 | 0.78+0.06 | GOL | France | superficial | Ropars et al., 2018 |
| CEC712 | 4 | 0.97+0.10 | ARS | France | superficial | Ropars et al., 2018 |
| CEC2023 | 8 | 1.06+0.08 | H014 | Guiana | commensal | Angebault et al., 2013 |
| CEC3613 | 8 | 1.16+0.05 | M47 | Morocco | commensal | Sdoudi et al., 2014 |
| CEC3634 | 8 | 1.40+0.190 | B54 | Brazil | commensal | Sdoudi et al., 2014 |
| CEC3663 | 8 | 0.87+0.06 | - | France | invasive | Ropars et al., 2018 |
| **CEC2018** | **9** | **0.96+0.14** | **APRURC3** | **France** | **superficial** | **Bougnoux et al., 2008** |
| CEC3668 | 9 | 0.71+0.05 | TNMURR20 | France | superficial | Bougnoux et al., 2008 |
| CEC3671 | 9 | 0.84+0.08 | TNMHCRC3 | France | invasive | Bougnoux et al., 2008 |
| CEC4261 | 9 | 1.05+0.12 | SOU2 | France | superficial | Garnaud et al., 2015 |
| CEC4692 | 9 | 0.84+0.04 | U820/ATCC MYA-3573 | - | invasive | - |
| CEC4693 | 9 | 0.84+0.07 | ATCC14053 / NIH 3172 | USA | invasive | - |
| CEC3616 | 10 | 1.13+0.03 | M15 | Morocco | commensal | Sdoudi et al., 2014 |
| CEC3711 | 10 | 0.87+0.08 | EGPURRM25 | France | superficial | Bougnoux et al., 2008 |
| CEC4024 | 10 | 1.25+0.05 | 303 | UK | invasive | - |
| CEC4660 | 10 | 0.88+0.07 | DESM70 | France | superficial | - |
| CEC3494 | 11 | 0.99+0.13 | C63 | Belgium | commensal | Bougnoux et al., 2006 |
| CEC3561 | 11 | 1.21+0.19 | C71 | Belgium | commensal | Bougnoux et al., 2006 |
| CEC3618 | 11 | 0.94+0.15 | C90 | Belgium | commensal | Bougnoux et al., 2006 |
| CEC3704 | 11 | 1.13+0.06 | EGPURRM9 | France | superficial | Bougnoux et al., 2008 |
| **CEC4492** | **11** | **1.06+0.10** | **-** | **unknown** | **food spoilage** | **Ropars et al., 2018** |
| CEC4510 | 11 | 0.94+0.09 | BLOV56 | France | superficial | - |
| CEC4525 | 11 | 1.06+0.15 | FLON07 | France | superficial | Ropars et al., 2018 |
| CEC5120 | 11 | 1.48+0.08 | UMIP 200.77 | France | superficial | - |
| CEC3537 | 12 | 0.90+0.08 | M26 | Morocco | commensal | Sdoudi et al., 2014 |
| CEC3685 | 12 | 0.90+0.08 | EGPHCRS1 | France | invasive | Bougnoux et al., 2008 |
| CEC3686 | 12 | 0.65+0.01 | EGPHCRS2 | France | invasive | Bougnoux et al., 2008 |
| CEC5114 | 12 | 0.60+0.07 | - | - | invasive | - |
| CEC4104 | 13 | 0.40+0.03 | CAAF2 | Senegal | superficial | Dieng et al., 2012 |
| **CEC4943** | **13** | **1.09+0.14** | **VPCI 891/P/12** | **India** | **superficial** | **Sharma et al., 2014** |
| CEC5020 | 13 | 0.46+0.05 | NCPF8946 | UK | superficial | Borman et al., 2013 |
| CEC5028 | 13 | 0.54+0.04 | NCPF8954 | UK | superficial | Borman et al., 2013 |
| CEC5029 | 13 | 0.54+0.06 | NCPF8955 | UK | superficial | Borman et al., 2013 |
| CEC3550 | 16 | 1.19+0.17 | X13 | France | starling | Bougnoux et al., 2004 |
| CEC3600 | 16 | 1.23+0.10 | X14 | France | starling | Bougnoux et al., 2004 |
| CEC3664 | 16 | 0.84+0.15 | LRBURRM1 | France | superficial | Bougnoux et al., 2008 |
| CEC2872 | 18 | 1.06+0.08 | B18 | S. Korea | invasive | Shin et al., 2011 |
| CEC2876 | 18 | 0.89+0.06 | A41 | S. Korea | invasive | Shin et al., 2011 |

**Supplemental Table 2: Growth Parameters Data.** Entries in bold face are representative isolates. Mean+SD (RSD) values are shown (n = 3). Parameters shown include saturation point (k), growth at start of culture (n0), growth rate (r), period at which population density reaches 1/2 of k(Inflection point, H) and Doubling time (h). Parameters were calculated based on Growthcurver package in R(52).

| **Clade** | **Isolate** | **k** | **n0** | **r** | **Inflection point (H)** | **Doubling time (h)** |
| --- | --- | --- | --- | --- | --- | --- |
| A | CEC3548 | 1.395+0.067(4.78) | 0.032+0.006(20.111) | 0.66+0.029(4.44) | 5.712+0.024(0.42) | 1.052+0.047(4.5) |
| A | CEC3715 | 1.352+0.056(4.18) | 0.044+0.004(8.491) | 0.709+0.016(2.25) | 4.782+0.033(0.7) | 0.978+0.022(2.22) |
| A | CEC4038 | 1.441+0.007(0.52) | 0.026+0.001(3.36) | 0.609+0.006(0.95) | 6.596+0.073(1.11) | 1.139+0.011(0.95) |
| B | CEC3708 | 1.424+0.017(1.21) | 0.035+0.003(8.144) | 0.62+0.013(2.14) | 5.956+0.094(1.57) | 1.119+0.024(2.16) |
| B | CEC4254 | 1.466+0.005(0.33) | 0.038+0.006(15.351) | 0.611+0.017(2.85) | 5.941+0.296(4.99) | 1.136+0.033(2.89) |
| C | CEC4039 | 1.444+0.024(1.7) | 0.047+0.003(6.88) | 0.521+0.011(2.06) | 6.498+0.041(0.63) | 1.33+0.027(2.04) |
| C | CEC5136* | 1.355+0.018(1.34) | 0.038+0.004(9.957) | 0.367+0.01(2.72) | 9.665+0.047(0.49) | 1.889+0.052(2.74) |
| D | CEC3707 | 1.457+0.012(0.79) | 0.031+0.002(6.85) | 0.555+0.013(2.27) | 6.918+0.057(0.82) | 1.248+0.028(2.27) |
| D | CEC4498 | 1.414+0.041(2.91) | 0.04+0.004(10.57) | 0.487+0.003(0.65) | 7.239+0.12(1.66) | 1.422+0.009(0.65) |
| E | CEC3712 | 1.472+0.036(2.48) | 0.041+0.001(1.765) | 0.587+0.005(0.82) | 6.034+0.097(1.61) | 1.18+0.01(0.83) |
| E | CEC4486 | 1.401+0.012(0.86) | 0.022+0.004(17.729) | 0.646+0.028(4.37) | 6.432+0.047(0.74) | 1.075+0.047(4.4) |
| C01 | CEC1424 | 1.457+0.05(3.41) | 0.041+0(0.754) | 0.571+0.012(2.06) | 6.218+0.175(2.81) | 1.214+0.025(2.04) |
| C01 | CEC3534 | 1.271+0.054(4.23) | 0.086+0.032(36.952) | 0.662+0.065(9.85) | 4.033+0.194(4.81) | 1.054+0.101(9.54) |
| C01 | CEC3621-1 | 1.44+0.024(1.69) | 0.039+0.002(4.734) | 0.587+0.006(1.04) | 6.117+0.01(0.16) | 1.181+0.012(1.03) |
| C01 | CEC3660 | 1.358+0.004(0.31) | 0.068+0.009(13.828) | 0.567+0.01(1.82) | 5.192+0.242(4.67) | 1.222+0.022(1.83) |
| C01 | CEC3672 | 1.452+0.029(2.03) | 0.05+0.003(6.403) | 0.53+0.015(2.77) | 6.286+0.05(0.8) | 1.308+0.036(2.77) |
| C01 | CEC3678 | 1.307+0.029(2.2) | 0.089+0.008(8.745) | 0.623+0.006(1.02) | 4.198+0.103(2.46) | 1.112+0.011(1.02) |
| C01 | CEC3679 | 1.455+0.028(1.92) | 0.047+0.003(6.981) | 0.54+0.014(2.63) | 6.291+0.04(0.64) | 1.284+0.033(2.59) |
| C01 | CEC4035 | 1.41+0.035(2.48) | 0.057+0.002(4.087) | 0.45+0.01(2.21) | 7.037+0.095(1.35) | 1.542+0.034(2.23) |
| C01 | CEC4256 | 1.452+0.035(2.43) | 0.03+0.002(6.873) | 0.545+0.017(3.07) | 7.054+0.128(1.81) | 1.272+0.039(3.06) |
| C01 | CEC4479 | 1.462+0.043(2.97) | 0.055+0.009(16.775) | 0.545+0.017(3.13) | 5.958+0.111(1.86) | 1.274+0.039(3.09) |
| C01 | CEC4489 | 1.367+0.078(5.71) | 0.059+0.012(19.914) | 0.583+0.03(5.22) | 5.338+0.145(2.72) | 1.19+0.061(5.13) |
| C01 | CEC4499 | 1.513+0.015(1) | 0.035+0.001(3.856) | 0.532+0.009(1.73) | 7.017+0.156(2.22) | 1.303+0.023(1.75) |
| C01 | CEC4500 | 1.359+0.035(2.6) | 0.064+0.003(3.995) | 0.612+0.015(2.45) | 4.909+0.113(2.29) | 1.132+0.027(2.42) |
| C01 | CEC4512 | 1.296+0.048(3.67) | 0.06+0.01(16.075) | 0.583+0.017(3) | 5.214+0.106(2.03) | 1.189+0.035(2.95) |
| C01 | CEC5132 | 1.423+0.017(1.17) | 0.062+0.002(3.985) | 0.554+0.005(0.85) | 5.588+0.048(0.87) | 1.252+0.011(0.85) |
| **C01** | **SC5314** | **1.372+0.027(1.99)** | **0.052+0.007(12.922)** | **0.618+0.027(4.43)** | **5.248+0.037(0.71)** | **1.123+0.05(4.49)** |
| C02 | CEC2021 | 1.47+0.048(3.27) | 0.032+0.004(11.527) | 0.568+0.014(2.53) | 6.717+0.043(0.63) | 1.221+0.031(2.57) |
| C02 | CEC3531 | 1.397+0.022(1.59) | 0.043+0.003(7.129) | 0.563+0.006(1.12) | 6.118+0.068(1.11) | 1.231+0.014(1.13) |
| C02 | CEC3549 | 1.39+0.044(3.17) | 0.039+0.007(18.87) | 0.598+0.012(2.05) | 5.963+0.164(2.74) | 1.159+0.023(2.03) |
| C02 | CEC3558 | 1.51+0.071(4.67) | 0.081+0.005(6.786) | 0.233+0.001(0.36) | 12.351+0.083(0.68) | 2.978+0.011(0.36) |
| C02 | CEC3615 | 1.369+0.007(0.52) | 0.036+0.006(16.508) | 0.61+0.034(5.54) | 5.915+0.05(0.84) | 1.138+0.063(5.53) |
| **C02** | **CEC3669** | **1.372+0.017(1.22)** | **0.034+0.004(11.278)** | **0.642+0.009(1.37)** | **5.731+0.092(1.6)** | **1.079+0.015(1.37)** |
| C02 | CEC4482 | 1.341+0.055(4.13) | 0.026+0.009(33.604) | 0.691+0.063(9.1) | 5.731+0.051(0.89) | 1.008+0.088(8.74) |
| C02 | CEC4945 | 1.448+0.02(1.4) | 0.05+0.002(4.589) | 0.55+0.013(2.32) | 6.073+0.16(2.64) | 1.261+0.03(2.34) |
| C03 | CEC1289 | 1.273+0.044(3.49) | 0.064+0.01(15.615) | 0.729+0.016(2.22) | 4.042+0.175(4.34) | 0.95+0.021(2.21) |
| C03 | CEC3554 | 1.351+0.059(4.38) | 0.034+0.004(12.697) | 0.641+0.021(3.25) | 5.695+0.057(1) | 1.081+0.035(3.28) |
| C03 | CEC3557 | 1.517+0.038(2.51) | 0.024+0.001(3.792) | 0.561+0.009(1.6) | 7.4+0.11(1.49) | 1.236+0.02(1.61) |
| C03 | CEC3637 | 1.404+0.009(0.67) | 0.049+0.005(9.55) | 0.653+0.019(2.98) | 5.092+0.025(0.5) | 1.063+0.032(2.98) |
| C03 | CEC3681 | 1.396+0.023(1.67) | 0.052+0.006(10.639) | 0.585+0.033(5.65) | 5.562+0.121(2.18) | 1.187+0.066(5.56) |
| C03 | G3 | 1.414+0.093(6.6) | 0.019+0.004(18.978) | 0.65+0.012(1.84) | 6.627+0.108(1.63) | 1.067+0.02(1.84) |
| C04 | CEC1427 | 1.407+0.013(0.92) | 0.042+0.003(6.269) | 0.64+0.012(1.8) | 5.435+0.005(0.09) | 1.083+0.019(1.8) |
| C04 | CEC1492 | 1.419+0.01(0.73) | 0.056+0.003(4.994) | 0.565+0.015(2.65) | 5.65+0.207(3.67) | 1.228+0.032(2.61) |
| C04 | CEC2022 | 1.402+0.022(1.54) | 0.03+0.002(6.348) | 0.615+0.008(1.23) | 6.207+0.1(1.61) | 1.128+0.014(1.22) |
| C04 | CEC3530 | 1.422+0.053(3.74) | 0.034+0.003(9.973) | 0.631+0.013(2.07) | 5.868+0.013(0.23) | 1.099+0.023(2.06) |
| C04 | CEC3536 | 1.366+0.059(4.33) | 0.035+0.008(23.055) | 0.665+0.039(5.83) | 5.501+0.169(3.07) | 1.044+0.062(5.94) |
| C04 | CEC3551 | 1.36+0.021(1.52) | 0.086+0.01(12.201) | 0.606+0.041(6.78) | 4.468+0.059(1.31) | 1.147+0.076(6.65) |
| C04 | CEC3602 | 1.388+0.023(1.64) | 0.074+0.005(7.234) | 0.608+0.016(2.65) | 4.732+0.031(0.65) | 1.141+0.03(2.66) |
| C04 | CEC3607 | 1.375+0.061(4.43) | 0.035+0.008(22.548) | 0.701+0.033(4.78) | 5.235+0.022(0.42) | 0.991+0.047(4.78) |
| C04 | CEC3610 | 1.313+0.036(2.72) | 0.049+0.005(9.478) | 0.683+0.012(1.81) | 4.769+0.106(2.22) | 1.015+0.019(1.82) |
| **C04** | **CEC3675** | **1.357+0.039(2.84)** | **0.03+0.007(24.686)** | **0.669+0.041(6.12)** | **5.716+0.086(1.51)** | **1.039+0.066(6.34)** |
| C04 | CEC3676 | 1.433+0.065(4.56) | 0.023+0.006(25.219) | 0.638+0.028(4.35) | 6.482+0.069(1.07) | 1.087+0.047(4.36) |
| C04 | CEC4497 | 1.425+0.033(2.33) | 0.033+0.003(7.761) | 0.636+0.014(2.17) | 5.885+0.04(0.69) | 1.091+0.024(2.18) |
| C04 | CEC708 | 1.387+0.073(5.24) | 0.044+0.005(12.087) | 0.658+0.029(4.39) | 5.207+0.119(2.29) | 1.055+0.046(4.39) |
| C04 | CEC712 | 1.382+0.023(1.64) | 0.026+0.005(18.245) | 0.687+0.02(2.91) | 5.769+0.107(1.86) | 1.009+0.029(2.92) |
| C08 | CEC2023 | 1.52+0.043(2.82) | 0.027+0.002(9.099) | 0.603+0.012(2.05) | 6.68+0.062(0.92) | 1.15+0.023(2.04) |
| C08 | CEC3613 | 1.446+0.018(1.22) | 0.037+0.006(16.247) | 0.59+0.029(4.95) | 6.159+0.234(3.79) | 1.176+0.059(5.04) |
| C08 | CEC3634 | 1.394+0.039(2.77) | 0.043+0.004(8.267) | 0.714+0.019(2.64) | 4.84+0.025(0.51) | 0.971+0.026(2.68) |
| C08 | CEC3663-2 | 1.488+0.031(2.06) | 0.032+0.003(7.764) | 0.552+0.01(1.88) | 6.89+0.023(0.33) | 1.256+0.023(1.86) |
| **C09** | **CEC2018** | **1.378+0.054(3.93)** | **0.054+0.012(23.034)** | **0.634+0.037(5.77)** | **5.077+0.051(1)** | **1.095+0.062(5.7)** |
| C09 | CEC3668 | 1.457+0.049(3.34) | 0.051+0.009(16.977) | 0.526+0.03(5.68) | 6.337+0.062(0.99) | 1.321+0.073(5.52) |
| C09 | CEC3671 | 1.425+0.046(3.26) | 0.042+0.01(25.102) | 0.585+0.012(2.08) | 6.016+0.288(4.79) | 1.185+0.024(2.06) |
| C09 | CEC4261 | 1.346+0.02(1.47) | 0.039+0.007(18.108) | 0.648+0.02(3.01) | 5.443+0.21(3.86) | 1.07+0.032(3.03) |
| C09 | CEC4692 | 1.318+0.072(5.44) | 0.064+0.015(23.899) | 0.594+0.045(7.61) | 5.039+0.07(1.39) | 1.172+0.093(7.92) |
| C09 | CEC4693 | 1.461+0.009(0.63) | 0.037+0.002(5.081) | 0.553+0.011(2.05) | 6.582+0.057(0.86) | 1.253+0.026(2.07) |
| C10 | CEC3616 | 1.439+0.014(0.99) | 0.066+0.004(5.425) | 0.567+0.013(2.23) | 5.358+0.012(0.22) | 1.224+0.027(2.2) |
| C10 | CEC3711 | 1.362+0.084(6.2) | 0.047+0.009(19.13) | 0.613+0.028(4.57) | 5.443+0.095(1.75) | 1.133+0.052(4.55) |
| C10 | CEC4024 | 1.398+0.019(1.36) | 0.054+0.01(18.615) | 0.55+0.043(7.75) | 5.881+0.134(2.29) | 1.266+0.102(8.08) |
| C10 | CEC4660 | 1.458+0.008(0.58) | 0.038+0.002(4.654) | 0.556+0.008(1.44) | 6.508+0.032(0.49) | 1.248+0.018(1.45) |
| C11 | CEC3494 | 1.465+0.051(3.46) | 0.023+0.005(22.621) | 0.584+0.033(5.63) | 7.103+0.057(0.81) | 1.189+0.066(5.56) |
| C11 | CEC3561 | 1.419+0.056(3.95) | 0.031+0.004(12.103) | 0.548+0.006(1.16) | 6.957+0.089(1.28) | 1.266+0.015(1.17) |
| C11 | CEC3618 | 1.406+0.058(4.11) | 0.029+0.002(8.243) | 0.605+0.005(0.79) | 6.363+0.086(1.35) | 1.145+0.009(0.79) |
| C11 | CEC3704 | 1.481+0.026(1.73) | 0.026+0.003(10.404) | 0.639+0.016(2.45) | 6.317+0.098(1.55) | 1.085+0.026(2.42) |
| **C11** | **CEC4492** | **1.353+0.024(1.74)** | **0.097+0.006(6.682)** | **0.643+0.003(0.51)** | **3.995+0.098(2.46)** | **1.079+0.006(0.51)** |
| C11 | CEC4510 | 1.226+0.052(4.28) | 0.031+0.009(30.305) | 0.509+0.031(6.16) | 7.222+0.207(2.86) | 1.366+0.086(6.3) |
| C11 | CEC4525 | 1.356+0.062(4.57) | 0.024+0.004(15.592) | 0.646+0.03(4.7) | 6.268+0.092(1.47) | 1.075+0.05(4.65) |
| C11 | CEC5120 | 1.376+0.027(1.95) | 0.05+0.019(38.505) | 0.565+0.062(10.99) | 5.885+0.109(1.85) | 1.237+0.137(11.06) |
| C12 | CEC3537 | 1.492+0.034(2.25) | 0.02+0.002(10.936) | 0.631+0.015(2.38) | 6.838+0.064(0.93) | 1.099+0.026(2.41) |
| C12 | CEC3685 | 1.466+0.037(2.54) | 0.029+0.01(33.78) | 0.616+0.034(5.46) | 6.418+0.273(4.26) | 1.127+0.06(5.3) |
| C12 | CEC3686 | 1.352+0.018(1.32) | 0.041+0.005(12.225) | 0.524+0.013(2.51) | 6.619+0.06(0.91) | 1.323+0.034(2.54) |
| C12 | CEC5114 | 1.404+0.04(2.85) | 0.033+0.002(6.682) | 0.44+0.004(1) | 8.438+0.022(0.26) | 1.574+0.016(1) |
| C13 | CEC4104 | 1.411+0.066(4.71) | 0.022+0.005(22.57) | 0.434+0.018(4.11) | 9.623+0.028(0.29) | 1.599+0.067(4.2) |
| **C13** | **CEC4943** | **1.218+0.029(2.35)** | **0.082+0.012(15.311)** | **0.404+0.032(7.95)** | **6.546+0.18(2.74)** | **1.722+0.134(7.78)** |
| C13 | CEC5020 | 1.352+0.008(0.56) | 0.075+0.003(3.353) | 0.353+0.014(4.09) | 8.028+0.257(3.2) | 1.966+0.082(4.19) |
| C13 | CEC5028 | 1.296+0.039(3.02) | 0.083+0.024(29.104) | 0.427+0.051(12) | 6.335+0.158(2.49) | 1.639+0.21(12.79) |
| C13 | CEC5029 | 1.392+0.044(3.13) | 0.058+0.01(17.643) | 0.385+0.017(4.3) | 8.195+0.13(1.59) | 1.804+0.078(4.35) |
| C16 | CEC3550 | 1.38+0.062(4.52) | 0.071+0.001(1.714) | 0.574+0.02(3.49) | 5.071+0.068(1.34) | 1.208+0.043(3.56) |
| C16 | CEC3600 | 1.339+0.046(3.41) | 0.08+0.009(10.62) | 0.609+0.032(5.3) | 4.531+0.04(0.89) | 1.141+0.059(5.19) |
| C16 | CEC3664 | 1.527+0.034(2.22) | 0.03+0.001(2.914) | 0.571+0.006(0.98) | 6.851+0.029(0.43) | 1.213+0.012(0.99) |
| C18 | CEC2872 | 1.339+0.056(4.21) | 0.042+0.009(22.27) | 0.695+0.041(5.92) | 4.945+0.027(0.54) | 0.999+0.059(5.9) |
| C18 | CEC2876 | 1.418+0.015(1.057) | 0.04+0.002(5.584) | 0.629+0.015(2.366) | 5.62+0.107(1.91) | 1.102+0.026(2.372) |
| NC | CEC3619 | 1.238+0.036(2.901) | 0.048+0.008(15.955) | 0.38+0.03(8.006) | 8.509+0.345(4.057) | 1.832+0.153(8.363) |
| NC | CEC3665 | 1.372+0.007(0.486) | 0.107+0.034(31.812) | 0.608+0.053(8.676) | 4.113+0.214(5.207) | 1.147+0.103(8.981) |
| NC | CEC4485 | 1.422+0.047(3.317) | 0.062+0.007(11.955) | 0.536+0.021(3.933) | 5.783+0.095(1.645) | 1.294+0.051(3.922) |
| NC | CEC4502 | 1.508+0.025(1.663) | 0.043+0(1.104) | 0.57+0.01(1.797) | 6.211+0.067(1.086) | 1.217+0.022(1.816) |
| NC | CEC723 | 1.463+0.023(1.574) | 0.033+0.002(7.367) | 0.583+0.013(2.175) | 6.461+0.02(0.317) | 1.188+0.026(2.175) |

*CEC5136 did not grow in both trial and batch cultures

**Supplemental Table 3: Metabolite Concentrations (µM) in representative isolates.** Except for glucose in Clade 13, metabolite concentrations have RSD < 30%. Mean+SD (RSD) values of metabolite concentration in all seven batches are shown (n = 14, except CEC3675 with n = 12).

| **Isolate** | **SC5314** | **CEC3669** | **CEC3675** | **CEC2018** | **CEC4492** | **CEC4943** | **YPD** |
| --- | --- | --- | --- | --- | --- | --- | --- |
| **Clade** | **C01** | **C02** | **C04** | **C09** | **C11** | **C13** | **Culture Media** |
| **Acetate** | 1443.79+181.24(12.55) | 1459.94+123.26(8.44) | 1641.18+88.89(5.42) | 1531.76+183.24(11.96) | 1416.28+54.53(3.85) | 908.43+44.87(4.94) | 1090.53+72.25(6.63) |
| **Adenosine** | 209.19+21.95(10.49) | 265.71+12.42(4.68) | 223.5+9(4.03) | 257.01+17.42(6.78) | 248.5+8.61(3.46) | 201.56+23.75(11.78) | 313.89+12.43(3.96) |
| **Alanine** | 6708.25+364.08(5.43) | 6631.24+260.16(3.92) | 6564.73+180.35(2.75) | 6527.52+286.3(4.39) | 6531.98+169.12(2.59) | 6382.41+316.8(4.96) | 6512.59+216.8(3.33) |
| **Arginine** | 2991.77+313.31(10.47) | 2866.67+232.67(8.12) | 3052.96+256.92(8.42) | 2915.65+183.61(6.3) | 2873.92+184.11(6.41) | 2785.79+183.02(6.57) | 3126.01+594.62(19.02) |
| **Asparagine** | 1706.19+126.18(7.4) | 1634.06+56.03(3.43) | 1687.02+56.95(3.38) | 1705.79+87.63(5.14) | 1660.79+52.72(3.17) | 1481.48+102.07(6.89) | 1934.2+92.79(4.8) |
| **Aspartate** | 2074.96+133.51(6.43) | 2026.19+74.73(3.69) | 2053.95+74.84(3.64) | 2038.04+98.59(4.84) | 1997.56+47.18(2.36) | 1890.12+94.89(5.02) | 2057.8+51.67(2.51) |
| **Betaine** | 998.8+146.11(14.63) | 1053.37+78.98(7.5) | 1052.82+51.61(4.9) | 1065.94+87.71(8.23) | 1059.36+48.12(4.54) | 967.86+73.04(7.55) | 1270.79+92.74(7.3) |
| **Choline** | 25.61+7.06(27.57) | 19.73+4.11(20.85) | 21.37+3.89(18.21) | 31.08+4.82(15.5) | 19.64+4.06(20.7) | 35.91+8.34(23.21) | 54.7+6.42(11.74) |
| **D-(+)-malic acid** | 440.59+93.58(21.24) | 500.09+64.98(12.99) | 475.02+81.21(17.1) | 463.31+75.59(16.32) | 456.72+59.07(12.93) | 584.21+98.2(16.81) | 353.84+59.24(16.74) |
| **Ethanol** | 38190.57+8299.53(21.73) | 35531.89+5727.06(16.12) | 33264.28+7998.68(24.05) | 35797.91+8222.56(22.97) | 35013.61+6705.22(19.15) | 42953.42+6532.27(15.21) | 11052.31+2213.19(20.02) |
| **Formate** | 366.66+21.19(5.78) | 328.68+17.34(5.28) | 347.55+16.3(4.69) | 295.21+18.74(6.35) | 313.29+15.63(4.99) | 409.75+27.35(6.68) | 226.23+10.41(4.6) |
| **Fumarate** | 64.64+4.82(7.46) | 58.99+4.41(7.48) | 77.13+4(5.18) | 45.74+4.32(9.44) | 47.27+3.78(7.99) | 127.06+24.1(18.97) |  |
| **Glucose** | 44196.5+5515.21(12.48) | 48288.53+3053.49(6.32) | 43920.28+2282.26(5.2) | 48528.84+5600.19(11.54) | 49278.26+2538.31(5.15) | 27587.89+8814.66(31.95) | 95093.46+3266.71(3.44) |
| **Glutamate** | 5502.44+438.03(7.96) | 5452.84+214.41(3.93) | 5508.43+175(3.18) | 5487.32+258.34(4.71) | 5436.26+180.31(3.32) | 5135.43+347.88(6.77) | 5624.49+224.25(3.99) |
| **Glycerol** | 3991.81+715.18(17.92) | 4115.27+631.81(15.35) | 4462.93+231.73(5.19) | 4032.78+782.59(19.41) | 3546.58+441.74(12.46) | 7430.09+849.31(11.43) | 1730.31+302.05(17.46) |
| **Glycine** | 2315.52+124.11(5.36) | 2280.59+84.51(3.71) | 2254.83+63.68(2.82) | 2245.06+91.98(4.1) | 2270.13+61.34(2.7) | 2208.89+104.14(4.71) | 2202.75+66.72(3.03) |
| **Guanosine** | 59.94+7.1(11.85) | 57.02+5.59(9.8) | 52.13+3.99(7.66) | 62.21+5.44(8.74) | 53.7+2.77(5.17) | 49.56+4.55(9.19) | 108.76+7.86(7.23) |
| **Histidine** | 1099.37+74.13(6.74) | 1093.04+41.72(3.82) | 1104.18+36.36(3.29) | 1095.49+51.86(4.73) | 1092.32+52.75(4.83) | 1052.99+58.24(5.53) | 1089.02+36.81(3.38) |
| **Isoleucine** | 3169.5+204.03(6.44) | 3112.83+111.86(3.59) | 3064.53+79.55(2.6) | 3116.9+117.81(3.78) | 3116.66+72.81(2.34) | 3008.54+138.85(4.62) | 3220.69+115.61(3.59) |
| **Leucine** | 8513.78+603.34(7.09) | 8455.51+308.6(3.65) | 8376.58+283.41(3.38) | 8385.91+384.87(4.59) | 8340.32+240.87(2.89) | 8146.64+411.8(5.05) | 8722.58+236.33(2.71) |
| **Lysine** | 6093.65+529.66(8.69) | 5968.69+261.95(4.39) | 5901.56+190.29(3.22) | 5904.66+306.31(5.19) | 6003.78+162.87(2.71) | 5840.85+319.43(5.47) | 5738.13+233.22(4.06) |
| **Methionine** | 1832.38+107.61(5.87) | 1783.54+103.63(5.81) | 1792.94+55.92(3.12) | 1805.66+77.83(4.31) | 1784.95+51.3(2.87) | 1710.6+98.61(5.76) | 1765.34+65.8(3.73) |
| **Niacinamide** | 31.36+3.03(9.67) | 36.48+4.18(11.47) | 32.39+3.67(11.34) | 37.05+3.87(10.45) | 36.56+4.49(12.28) | 30.95+4.73(15.28) | 61.21+6.75(11.03) |
| **Nicotinate** | 52.21+3.73(7.15) | 51.44+3.88(7.54) | 51.49+3.48(6.77) | 46.51+3.43(7.38) | 50.32+4.55(9.04) | 49.36+4.41(8.94) | 43.9+6.51(14.82) |
| **Ornithine** | 212.15+25.69(12.11) | 201.43+31.99(15.88) | 223.19+31.97(14.32) | 204.31+32.78(16.05) | 224.24+47.05(20.98) | 208.8+28.93(13.85) | 203.49+32.05(15.75) |
| **Phenylalanine** | 3628.7+290.69(8.01) | 3632.43+168.28(4.63) | 3625.03+83.86(2.31) | 3588.66+165.26(4.6) | 3544.57+110.2(3.11) | 3519.59+172.6(4.9) | 3703.04+106.38(2.87) |
| **Proline** | 1577.54+126.58(8.02) | 1543.36+52.01(3.37) | 1520.4+57.01(3.75) | 1565.14+111.7(7.14) | 1578.16+86.93(5.51) | 1532.27+91.12(5.95) | 1848.22+169.41(9.17) |
| **Pyroglutamate** | 3675.28+256.94(6.99) | 3539.96+151.74(4.29) | 3447.36+141.04(4.09) | 3496.05+171.45(4.9) | 3541.41+105.22(2.97) | 3466.09+225.73(6.51) | 3622.34+187.76(5.18) |
| **Pyruvate** | 690.06+56.22(8.15) | 490.6+55.09(11.23) | 600.32+26.42(4.4) | 459.36+58.01(12.63) | 418.65+36.13(8.63) | 1011.54+128.86(12.74) | 150.91+12.36(8.19) |
| **Serine** | 3417.09+286.82(8.39) | 3278.93+162.09(4.94) | 3322.7+124.44(3.75) | 3373.71+165.87(4.92) | 3331.24+99.39(2.98) | 3094.21+190.99(6.17) | 3630.53+126.46(3.48) |
| **Succinate** | 895.51+81.83(9.14) | 828.89+46.21(5.57) | 884.63+46.51(5.26) | 822.04+54.45(6.62) | 821.97+55.74(6.78) | 909.33+67.22(7.39) | 680.13+49.46(7.27) |
| **Threonine** | 2662.25+205.97(7.74) | 2512.46+108.75(4.33) | 2548.76+94.68(3.71) | 2574.72+158.69(6.16) | 2562.38+90.99(3.55) | 2291.34+163.08(7.12) | 2882.46+113.3(3.93) |
| **Trehalose** | 1083.97+68.62(6.33) | 1048.71+60.83(5.8) | 1030.68+38.65(3.75) | 987.05+51.65(5.23) | 1038.04+40.74(3.92) | 1255.96+105.27(8.38) | 1218.36+60.84(4.99) |
| **Tryptophan** | 1119.88+113.82(10.16) | 1140.68+60.05(5.26) | 1142.11+60.97(5.34) | 1119.87+88.27(7.88) | 1096.46+81.86(7.47) | 1071.02+73.39(6.85) | 1155.81+92.28(7.98) |
| **Tyrosine** | 1071.61+60.76(5.67) | 1046.55+54.44(5.2) | 1045.83+35.61(3.4) | 1021.97+30.28(2.96) | 1030.97+29.77(2.89) | 1026.74+50.87(4.95) | 1050.54+29.15(2.77) |
| **Uracil** | 169.59+7.12(4.2) | 167.21+7.08(4.23) | 166.48+4.4(2.64) | 168.06+7.2(4.28) | 167.75+4.81(2.87) | 169.76+7.05(4.15) | 152.94+4.82(3.15) |
| **Uridine** | 30.31+4.85(16.01) | 29.36+4.67(15.89) | 27.13+3.28(12.1) | 28.44+3.38(11.87) | 28.01+3.4(12.14) | 27.16+4.48(16.51) | 26.63+6.25(23.47) |
| **Valine** | 4339.99+249(5.74) | 4279.39+166.08(3.88) | 4240.82+126.62(2.99) | 4258.36+174.13(4.09) | 4277.69+92.26(2.16) | 4187.59+188.38(4.5) | 4308.06+149.35(3.47) |
| **sn-Glycero-3-phosphocholine** | 269.32+44.15(16.39) | 311.76+47.63(15.28) | 264.9+42.12(15.9) | 302.64+45.36(14.99) | 305.06+19.84(6.5) | 269.64+37.04(13.74) | 393.17+84.89(21.59) |

**Supplemental Table 4. Comparison of normalization strategies for metabolite concentrations.** Min-Max normalization (metabolite concentrations adjusted based on maximum and minimum OD) and ∆ concentration (metabolite concentrations in the media control was subtracted from that of the samples and OD was normalized to 1. RSD from the largest clades are shown. RSD > 30% are in bold and italicized.

| **Metabolite** | **Clade 01** | | | **Clade 03** | | | **Clade 04** | | | **Clade 09** | | | **Clade 11** | | | **Clade 13** | | |
| --- | --- | --- | --- | --- | --- | --- | --- | --- | --- | --- | --- | --- | --- | --- | --- | --- | --- | --- |
|  | **Raw** | **Min-Max** | **∆ conc.** | **Raw** | **Min-Max** | **∆ conc.** | **Raw** | **Min-Max** | **∆ conc.** | **Raw** | **Min-Max** | **∆ conc.** | **Raw** | **Min-Max** | **∆ conc.** | **Raw** | **Min-Max** | **∆ conc.** |
| **Acetate** | 16.70 | ***35.17*** | ***64.22*** | 23.51 | ***32.12*** | ***57.47*** | 12.58 | 20.04 | ***40.05*** | 9.82 | 20.26 | ***36.78*** | 19.82 | 24.06 | ***97.96*** | 23.22 | ***50.83*** | ***115.72*** |
| **Adenosine** | 12.18 | ***33.50*** | ***43.38*** | 20.94 | 25.85 | ***56.71*** | 10.62 | 19.70 | ***37.90*** | 8.97 | 19.64 | ***40.29*** | 8.17 | 18.46 | 28.09 | 17.50 | ***49.12*** | ***42.22*** |
| **Alanine** | 4.37 | 25.07 | ***439.47*** | 20.06 | 24.45 | ***271.20*** | 3.54 | 14.73 | ***465.83*** | 5.51 | 16.37 | ***235.02*** | 3.65 | 15.69 | ***414.06*** | 3.81 | ***39.12*** | ***847.20*** |
| **Arginine** | 10.70 | 25.30 | ***142.94*** | 21.92 | 24.06 | ***249.90*** | 9.19 | 16.73 | ***176.46*** | 10.19 | 17.51 | ***366.87*** | 6.65 | 14.93 | ***121.45*** | 12.13 | ***39.18*** | ***230.09*** |
| **Asparagine** | 4.99 | 27.26 | ***62.70*** | 20.28 | 24.85 | ***57.01*** | 4.71 | 16.40 | ***33.81*** | 5.89 | 17.37 | ***48.07*** | 4.41 | 16.77 | ***31.92*** | 8.93 | ***43.68*** | ***32.62*** |
| **Aspartate** | 4.40 | 25.71 | ***955.45*** | 20.17 | 24.66 | ***887.51*** | 4.01 | 14.87 | ***229.18*** | 5.61 | 16.53 | ***500.21*** | 3.63 | 15.94 | ***136.80*** | 5.18 | ***40.65*** | ***202.93*** |
| **Betaine** | 9.24 | 27.05 | ***53.04*** | 19.97 | 24.47 | ***42.80*** | 6.04 | 16.91 | ***33.66*** | 9.25 | 18.62 | ***53.80*** | 7.51 | 19.01 | ***31.35*** | 7.97 | ***42.38*** | ***43.25*** |
| **Choline** | 25.80 | ***41.23*** | ***40.97*** | 25.77 | ***56.34*** | ***64.06*** | ***30.83*** | ***38.71*** | ***38.02*** | 20.22 | ***30.59*** | ***40.87*** | 19.69 | 24.46 | 23.81 | 29.68 | ***59.15*** | ***637.67*** |
| **D-(+)-malic acid** | 19.00 | 29.28 | ***127.21*** | ***39.82*** | ***34.65*** | ***107.95*** | 18.29 | 19.81 | ***86.49*** | 18.28 | 19.29 | ***88.08*** | 17.72 | 24.81 | ***86.33*** | 19.04 | ***39.22*** | ***73.85*** |
| **Ethanol** | 21.17 | 26.32 | ***31.97*** | 29.83 | 26.89 | ***33.60*** | 19.98 | 24.95 | ***31.39*** | 20.50 | 24.14 | 29.76 | 24.24 | 23.31 | ***31.64*** | ***31.15*** | 29.87 | ***34.95*** |
| **Formate** | 13.94 | 23.02 | ***53.34*** | 20.15 | 22.65 | ***30.36*** | 7.54 | 14.56 | 27.75 | 7.08 | 15.38 | ***35.97*** | 6.96 | 17.96 | ***35.27*** | 17.38 | 27.40 | 25.68 |
| **Fumarate** | 26.52 | ***33.17*** | ***33.17*** | 28.46 | 28.56 | ***30.35*** | 16.03 | 21.98 | 21.98 | 25.59 | 29.94 | 29.94 | 27.50 | ***32.54*** | ***34.18*** | 34.84 | ***40.84*** | ***40.84*** |
| **Glucose** | 15.91 | ***36.59*** | ***17.55*** | 23.15 | ***32.87*** | 25.23 | 10.53 | 21.37 | 13.10 | 11.90 | 23.80 | 12.22 | 19.83 | 27.90 | ***17.46*** | 38.70 | ***61.98*** | 12.18 |
| **Glutamate** | 5.08 | 26.12 | ***487.45*** | 20.24 | 24.81 | ***276.58*** | 4.49 | 15.38 | ***113.87*** | 6.50 | 17.20 | ***3882.10*** | 4.55 | 15.49 | ***92.59*** | 6.04 | ***40.84*** | ***211.52*** |
| **Glycerol** | 20.24 | 26.66 | ***35.87*** | 24.59 | ***32.09*** | ***41.64*** | 16.65 | 21.75 | ***28.34*** | 18.72 | 15.02 | 25.39 | 20.35 | 23.55 | ***36.78*** | 29.20 | 23.37 | 24.69 |
| **Glycine** | 4.28 | 24.95 | ***185.23*** | 20.18 | 23.90 | ***197.00*** | 3.64 | 14.88 | ***299.90*** | 5.20 | 16.34 | ***157.08*** | 3.44 | 15.64 | ***129.41*** | 3.35 | ***38.26*** | ***291.28*** |
| **Guanosine** | 17.31 | **37.35** | ***36.16*** | 21.17 | 25.87 | ***45.04*** | 12.49 | 21.43 | 20.37 | 9.90 | 19.63 | 20.93 | 9.19 | 18.50 | 18.85 | 21.01 | ***52.16*** | 22.94 |
| **Histidine** | 4.65 | 25.35 | ***2638.66*** | 20.07 | 24.09 | ***1059.13*** | 3.91 | 14.87 | ***471.66*** | 6.11 | 16.01 | ***382.94*** | 4.49 | 15.56 | ***660.11*** | 4.21 | ***38.95*** | ***224.88*** |
| **Isoleucine** | 4.44 | 25.75 | ***257.95*** | 20.42 | 24.50 | ***457.24*** | 3.83 | 15.38 | ***95.64*** | 5.21 | 16.18 | ***375.18*** | 3.97 | 16.20 | ***131.57*** | 4.21 | ***40.14*** | ***179.22*** |
| **Lactate** | 25.21 | ***33.95*** | ***185.86*** | ***31.30*** | ***37.68*** | ***293.94*** | 25.96 | ***30.25*** | ***236.09*** | 24.23 | 28.52 | ***166.54*** | 25.04 | 26.19 | ***358.89*** | 25.94 | ***47.84*** | ***121.47*** |
| **Leucine** | 4.76 | 26.22 | ***309.06*** | 20.15 | 24.76 | ***278.57*** | 3.83 | 15.06 | ***100.60*** | 5.86 | 17.00 | ***374.49*** | 4.03 | 15.91 | ***116.42*** | 4.67 | ***40.44*** | ***265.98*** |
| **Lysine** | 5.10 | 25.13 | ***111.56*** | 20.10 | 25.26 | ***120.65*** | 4.23 | 14.85 | ***147.08*** | 5.34 | 15.79 | ***114.02*** | 3.88 | 15.76 | ***105.53*** | 4.09 | ***38.36*** | ***228.65*** |
| **Methionine** | 4.32 | 25.49 | ***151.39*** | 20.02 | 24.58 | ***143.75*** | 3.89 | 15.42 | ***324.67*** | 5.98 | 16.73 | ***130.70*** | 4.02 | 15.99 | ***249.84*** | 4.59 | ***39.96*** | ***235.15*** |
| **Niacinamide** | 14.04 | ***32.87*** | 29.72 | 23.58 | ***39.31*** | ***56.65*** | 12.09 | 21.23 | 28.11 | 11.09 | 21.65 | 27.80 | 16.63 | 22.55 | 23.96 | 18.78 | ***50.14*** | ***31.94*** |
| **Nicotinate** | 8.52 | 22.89 | ***99.02*** | 21.33 | 18.34 | ***160.70*** | 6.03 | 13.96 | ***79.11*** | 8.33 | 15.71 | ***110.17*** | 9.77 | 14.01 | ***78.70*** | 9.87 | ***36.02*** | ***227.01*** |
| **Ornithine** | 13.71 | 26.34 | ***1246.15*** | 23.39 | 25.06 | ***445.12*** | 14.48 | 19.03 | ***756.90*** | 18.50 | 22.95 | ***4193.17*** | 15.87 | 19.41 | ***361.44*** | 12.15 | ***39.96*** | ***3932.98*** |
| **Phenylalanine** | 5.00 | 25.61 | ***192.12*** | 19.92 | 25.09 | ***135.00*** | 3.82 | 15.13 | ***117.50*** | 5.99 | 16.70 | ***370.66*** | 3.99 | 16.04 | ***89.49*** | 5.02 | ***40.17*** | ***798.49*** |
| **Proline** | 8.44 | 28.48 | ***87.34*** | 20.21 | 24.81 | ***55.82*** | 5.23 | 16.98 | ***46.45*** | 7.17 | 18.15 | ***54.23*** | 4.35 | 16.60 | ***42.40*** | 6.28 | ***41.53*** | ***65.09*** |
| **Pyroglutamate** | 5.32 | 25.16 | ***221.04*** | 20.15 | 26.28 | ***312.68*** | 5.18 | 15.80 | ***163.41*** | 5.53 | 16.19 | ***461.24*** | 4.05 | 15.65 | ***171.90*** | 5.15 | ***39.27*** | ***230.13*** |
| **Pyruvate** | 26.35 | 17.70 | 21.26 | 28.69 | ***31.79*** | ***37.80*** | 17.63 | 23.60 | 29.41 | 18.14 | 21.80 | ***28.02*** | 21.53 | 22.89 | ***30.51*** | 51.66 | 12.32 | 28.10 |
| **Serine** | 5.52 | 26.99 | ***142.72*** | 20.24 | 25.69 | ***96.38*** | 5.01 | 15.77 | ***56.05*** | 6.93 | 17.75 | ***107.50*** | 4.74 | 17.08 | ***50.06*** | 7.69 | ***43.24*** | ***92.76*** |
| **Succinate** | 9.75 | 21.65 | ***71.16*** | 20.45 | 23.34 | ***53.49*** | 7.04 | 13.82 | ***42.30*** | 7.59 | 13.71 | ***56.60*** | 8.66 | 13.83 | ***42.09*** | 11.94 | ***31.85*** | ***49.42*** |
| **Threonine** | 5.27 | 26.72 | ***70.55*** | 20.39 | 25.42 | ***68.78*** | 5.20 | 16.66 | ***41.48*** | 6.84 | 17.84 | ***68.42*** | 4.84 | 17.08 | ***36.97*** | 8.54 | ***43.20*** | ***37.42*** |
| **Trehalose** | 7.83 | 27.28 | ***89.37*** | 20.99 | 24.22 | ***62.09*** | 6.17 | 16.23 | ***44.08*** | 9.09 | 19.41 | ***50.37*** | 9.57 | 18.84 | ***50.20*** | 6.68 | ***37.72*** | ***466.70*** |
| **Tryptophan** | 6.64 | 26.65 | ***325.76*** | 20.13 | 23.94 | ***341.53*** | 5.54 | 15.80 | ***138.30*** | 7.99 | 17.31 | ***854.75*** | 5.91 | 17.11 | ***171.87*** | 6.51 | ***40.93*** | ***4972.17*** |
| **Tyrosine** | 4.75 | 25.29 | ***1278.57*** | 20.17 | 24.38 | ***473.05*** | 4.86 | 15.38 | ***625.33*** | 5.99 | 16.70 | ***1235.21*** | 3.97 | 15.95 | ***722.13*** | 4.22 | ***39.33*** | ***590.27*** |
| **Uracil** | 5.41 | 25.17 | ***58.21*** | 20.13 | 20.47 | ***91.94*** | 3.92 | 14.11 | ***47.25*** | 4.75 | 14.89 | ***55.48*** | 7.03 | 15.69 | ***68.71*** | 4.90 | ***35.64*** | ***85.78*** |
| **Uridine** | 14.52 | ***31.43*** | ***172.19*** | 22.74 | ***32.58*** | ***286.72*** | 15.42 | 22.45 | ***449.58*** | 13.55 | 19.76 | ***129.03*** | 13.84 | 20.33 | ***875.73*** | 14.33 | ***41.19*** | ***522.03*** |
| **Valine** | 4.30 | 25.48 | ***3190.75*** | 20.26 | 24.47 | ***524.69*** | 3.80 | 15.01 | ***317.19*** | 5.34 | 16.50 | ***499.48*** | 3.85 | 16.25 | ***1128.34*** | 3.86 | ***39.44*** | ***1835.88*** |
| **sn-Glycero-3-phosphocholine** | 13.26 | ***34.02*** | ***141.44*** | 20.14 | 25.32 | ***82.22*** | 12.34 | 23.36 | ***78.38*** | 10.38 | 20.31 | ***70.04*** | 10.50 | 22.00 | ***106.95*** | 16.35 | ***48.20*** | ***189.46*** |

**Supplemental Table 5: Within-clade comparison of metabolites (fumarate and glycerol) most responsive to isolate label.** The table shows values for test of normality (Shapiro-Wilk) and equality of variance (Levene’s test). Normally distributed data with equal variance were analyzed with Fisher’s ANOVA while those with unequal variance were analyzed using Welch ANOVA. Kruskal Wallis Tests were carried out for datasets violating the assumption of normality. Significance values for isolate comparison were based in either mean for parametric (one-way ANOVA) or median for non-parametric (Kruskal Wallis) analysis. The computation for the test statistic and effect sizes are embedded in the R package used. Only the comparisons for largest clades are shown (n = 14 for representative isolates, except CEC3675 with n = 12; n = 6 for the rest except CEC3558 and CEC5120 with n = 4).

| Clade | Shapiro-Wilk | Levene’s test | Isolate comparison | Test Statistic | Effect size |
| --- | --- | --- | --- | --- | --- |
| Fumarate | | | | | |
| 01 | *p* = 0.005 |  | *p*< 0.001 | χ^2^_Kruskal-Wallis_= 96.23 | ε^2^_ordinal_ = 0.92 |
| 02 | *p* = 0.41 | *p* = 0.12 | *p*< 0.001 | *F*_Fisher_ = 54.96 | ω^2^*_p_* = 0.87 |
| 03 | *p* = 0.39 | *p* = 0.03 | *p*< 0.001 | *F*_Welch_ = 70.29 | ω^2^*_p_* = 0.95 |
| 04 | *p* = 0.26 | *p* = 0.05 | *p*< 0.001 | *F*_Fisher_ = 53.68 | ω^2^*_p_* = 0.88 |
| 09 | *p* = 0.02 |  | *p*< 0.001 | χ^2^_Kruskal-Wallis_= 37.89 | ε^2^_ordinal_ = 0.88 |
| 11 | *p* = 0.002 |  | *p*< 0.001 | χ^2^_Kruskal-Wallis_= 49.31 | ε^2^_ordinal_ = 0.93 |
| 13 | *p* = 0.22 | *p* = 0.01 | *p*< 0.001 | *F*_Welch_ = 56.00 | ω^2^*_p_* = 0.92 |
| Glycerol | | | | | |
| 01 | *p*< 0.001 |  | *p*< 0.001 | χ^2^_Kruskal-Wallis_= 53.16 | ε^2^_ordinal_ = 0.51 |
| 02 | *p*< 0.001 |  | *p*< 0.001 | χ^2^_Kruskal-Wallis_= 19.62 | ε^2^_ordinal_ = 0.37 |
| 03 | *p* = 0.94 | *p* = 0.90 | *p*< 0.001 | *F*_Fisher_ = 10.28 | ω^2^*_p_* = 0.56 |
| 04 | *p* = 0.03 |  | *p*< 0.001 | χ^2^_Kruskal-Wallis_= 55.81 | ε^2^_ordinal_ = 0.63 |
| 09 | *p* = 0.01 |  | *p* = 0.04 | χ^2^_Kruskal-Wallis_= 11.79 | ε^2^_ordinal_ = 0.27 |
| 11 | *p* = 0.19 | *p* = 0.11 | *p*< 0.001 | *F*_Fisher_ = 12.35 | ω^2^*_p_* = 0.60 |
| 13 | *p* = 0.001 |  | *p*< 0.001 | χ^2^_Kruskal-Wallis_= 28.77 | ε^2^_ordinal_ = 0.78 |

**Supplemental Table 6: Metabolite Class and Metabolic Pathways.** Table shows the metabolites annotated and quantified classified according to type and the pathways these metabolites are involved.

| **Metabolites** | **Metabolite Class** | **Metabolite Pathways** |
| --- | --- | --- |
| Acetate | Fatty acids | Pyruvate and Tricarboxylate metabolism |
| Adenosine | Nucleotides and nucleosides | Purine and Pyrimidine metabolism |
| Alanine | Amino acids | Alanine, Asparagine and Glutamate metabolism |
| Arginine | Amino acids, Amines |  |
| Asparagine | Amino acids | Alanine, Asparagine and Glutamate metabolism |
| Aspartate | Amino acids, Carboxylic acids | Alanine, Asparagine and Glutamate metabolism |
| Betaine | Amino acids | Glycine, Serine and Threonine metabolism |
| Choline | Alcohol and polyols, Amines | Lipid metabolism |
| Ethanol | Alcohol and polyols | Carbohydrate metabolism |
| Formate | Carboxylic acids | Pyruvate and Tricarboxylate metabolism |
| Fumarate | Carboxylic acids | Pyruvate and Tricarboxylate metabolism |
| Glucose | Monosacchrides and disaccharides | Carbohydrate metabolism |
| Glutamate | Amino acids, Carboxylic acids | Alanine, Asparagine and Glutamate metabolism |
| Glycerol | Alcohol and polyols | Lipid metabolism |
| Glycine | Amino acids | Glycine, Serine and Threonine metabolism |
| Guanosine | Nucleotides and nucleosides | Purine and Pyrimidine metabolism |
| Histidine | Aromatics | Histidine metabolism |
| Isoleucine | Amino acids | Valine, Leucine and Isoleucine metabolism |
| Leucine | Amino acids | Valine, Leucine and Isoleucine metabolism |
| Lysine | Amino acids, Amines | Lysine metabolism |
| Methionine | Amino acids, Sulfur compounds | Cysteine and Methionine metabolism |
| Niacinamide | Amides, Aromatics, Vitamins and cofactors | Arginine and Proline metabolism |
| Nicotinate | Amines, Aromatics, Carboxylic acids, Vitamins and cofactors |  |
| Ornithine | Amino acids, Amines | Urea cycle |
| Phenylalanine | Amino acids, Aromatics |  |
| Proline | Amino acids | Arginine and Proline metabolism |
| Pyroglutamate | Amino acids | Alanine, Asparagine and Glutamate metabolism |
| Pyruvate | Keto acids |  |
| Serine | Amino acids, Hydroxyacids | Glycine, Serine and Threonine metabolism |
| sn-Glycero-3-phosphocholine | Alcohol and polyols, Amines, Phosphates | Lipid metabolism |
| Succinate | Carboxylic acids | Pyruvate and Tricarboxylate metabolism |
| Threonine | Amino acids, Hydroxyacids | Glycine, Serine and Threonine metabolism |
| Trehalose | Monosacchrides and disaccharides | Carbohydrate metabolism |
| Tryptophan | Amino acids, Aromatics | Tryptophan metabolism |
| Tyrosine | Amino acids, Phenols | Phenylalanine and Tyrosine metabolism |
| Uracil | Nucleotides and nucleosides | Purine and Pyrimidine metabolism |
| Uridine | Nucleotides and nucleosides | Purine and Pyrimidine metabolism |
| Valine | Amino acids | Valine, Leucine and Isoleucine metabolism |

**Supplemental Table 7. Chemical Shift Values for the Annotation and Quantification of Metabolites.**

| **Metabolites** | **Chemical shift δ (ppm)** | |
| --- | --- | --- |
|  | **Identification** | **Quantification** |
| Acetate | 1.9 | 1.9 |
| Adenosine | 3.8, 3.9, 4.3, 4.4, 4.8, 6.1, 8.2, 8.3 | 8.3 |
| Alanine | 1.5, 3.8 | 1.5 |
| Arginine | 1.6, 1.7, 1.9, 1.9, 3.2, 3.8, 6.7, 7.2 | 3.2 |
| Asparagine | 2.9, 2.9, 4.0, 6.9, 7.6 | 2.9 |
| Aspartate | 2.7, 2.8, 3.9 | 2.8 |
| Betaine | 3.3, 3.9 | 3.9 |
| Choline | 3.2, 3.5, 4.1 | 3.2 |
| D-(+)-malic acid | 2.4, 2.7, 4.3 | 2.7 |
| Ethanol | 1.2, 3.7 | 1.2 |
| Formate | 8.4 | 8.4 |
| Fumarate | 6.5 | 6.5 |
| Glucose | 3.2, 3.4, 3.4, 3.5, 3.5, 3.5, 3.7, 3.7, 3.8, 3.8, 3.8, 3.9, 4.6, 5.2 | 5.2 |
| Glutamate | 2.0, 2.1, 2.3, 2.4, 3.8 | 2.4 |
| Glycerol | 3.6, 3.6, 3.8 | 3.6 |
| Glycine | 3.6 | 3.6 |
| Guanosine | 3.8, 3.9, 4.2, 4.4, 4.8, 5.9, 6.3, 8.0 | 6.3 |
| Histidine | 3.2, 3.2, 4.0, 7.1, 7.9 | 7.1 |
| Isoleucine | 0.9, 1.0, 1.3, 1.5, 2.0, 3.7 | 1.0 |
| Lactate | 1.3, 4.1 | - |
| Leucine | 0.9, 1.0, 1.7, 1.7, 1.7, 3.7 | 0.9, 1.0 |
| Lysine | 1.4, 1.5, 1.7, 1.9, 1.9, 3.0, 3.8 | 3.0 |
| Methionine | 2.1, 2.1, 2.2, 2.6, 3.9 | 2.1 |
| Niacinamide | 7.4, 7.6, 8.2, 8.2, 8.7, 8.9 | 8.7 |
| Nicotinate | 7.5, 8.2, 8.6, 8.9 | 8.6 |
| Ornithine | 1.7, 1.8, 1.9, 3.0, 3.8 | 3.0 |
| Phenylalanine | 3.1, 3.3, 4.0, 7.3, 7.4, 7.4 | 7.4 |
| Proline | 2.0, 2.0, 2.1, 2.3, 3.3, 3.4, 4.1 | 3.3 |
| Pyroglutamate | 2.0, 2.4, 2.4, 2.5, 4.2, 7.7 | 2.5 |
| Pyruvate | 2.4 | 2.4 |
| Serine | 3.8, 3.9, 4.0 | 3.9 |
| Succinate | 2.4 | 2.4 |
| Threonine | 1.3, 3.6, 4.1 | 1.3 |
| Trehalose | 3.4, 3.6, 3.8, 3.8, 3.8, 3.9, 5.2 | 5.2 |
| Tryptophan | 3.3, 3.5, 4.1, 7.2, 7.3, 7.3, 7.5, 7.7, 10.2 | 7.7 |
| Tyrosine | 3.0, 3.2, 3.9, 6.9, 7.2 | 6.9 |
| Uracil | 5.8, 7.5 | 5.8 |
| Uridine | 3.8, 3.9, 4.1, 4.2, 4.3, 59, 5.9, 7.9 | 5.9 |
| Valine | 1.0, 1.0, 2.3, 3.6 | 1.0 |
| sn-Glycero-3-phosphocholine | 3.2, 3.6, 3.7, 3.7, 3.9, 3.9, 3.9, 4.3 | 3.2 |

| 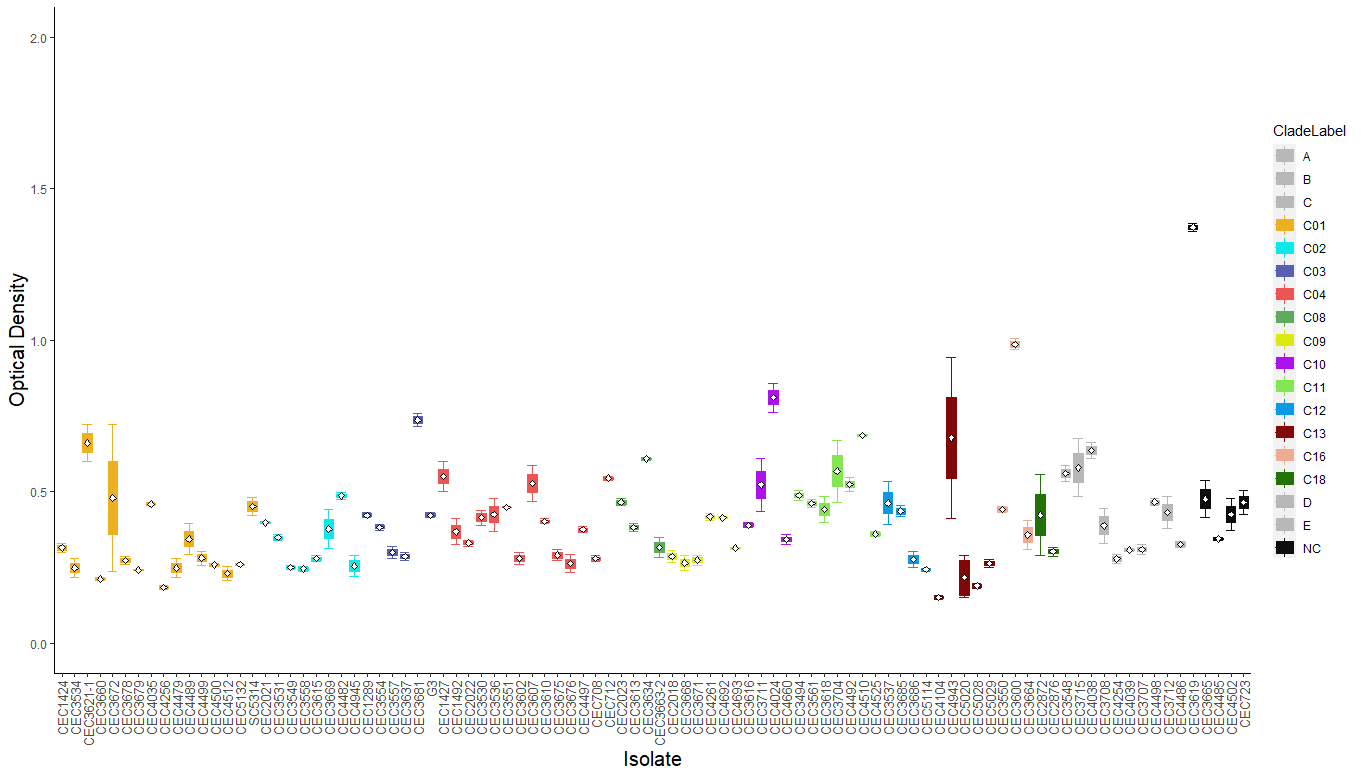 |
| --- |

**Supplemental Figure 1. Trial culture OD.** The culture was initiated at initial OD = 0.1 and done in duplicate. The data are indicated as mean (white diamond), interquartile range (IQR) (colored boxplots) and whiskers which correspond to 1.5 times IQR (n=2).


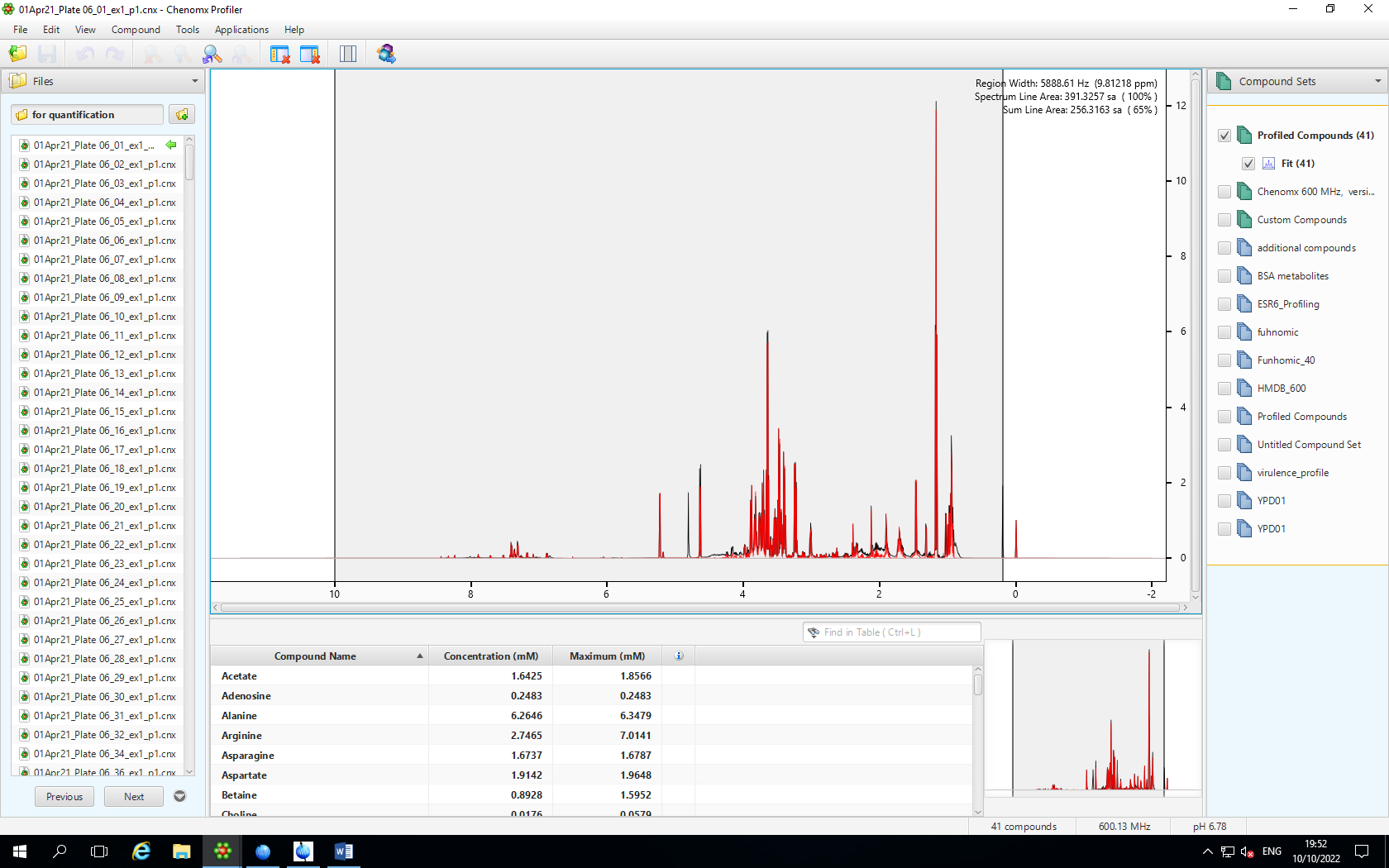


**Supplemental Figure 2. The NMR spectrum with annotated and quantified regions (in red) using Chenomx NMR suite.** The annotated and quantified spectra correspond to around 65% of the spectral region covering 0.2 to 10 ppm.

| 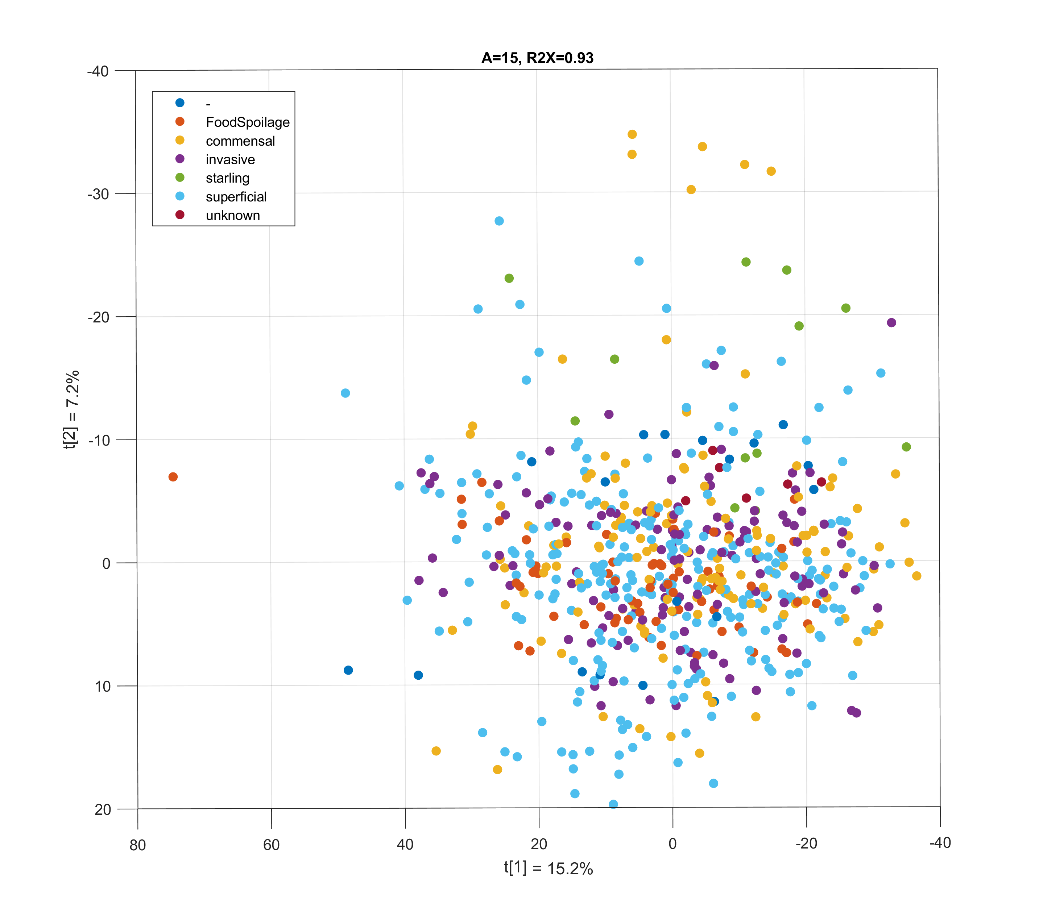  A |
| --- |
| 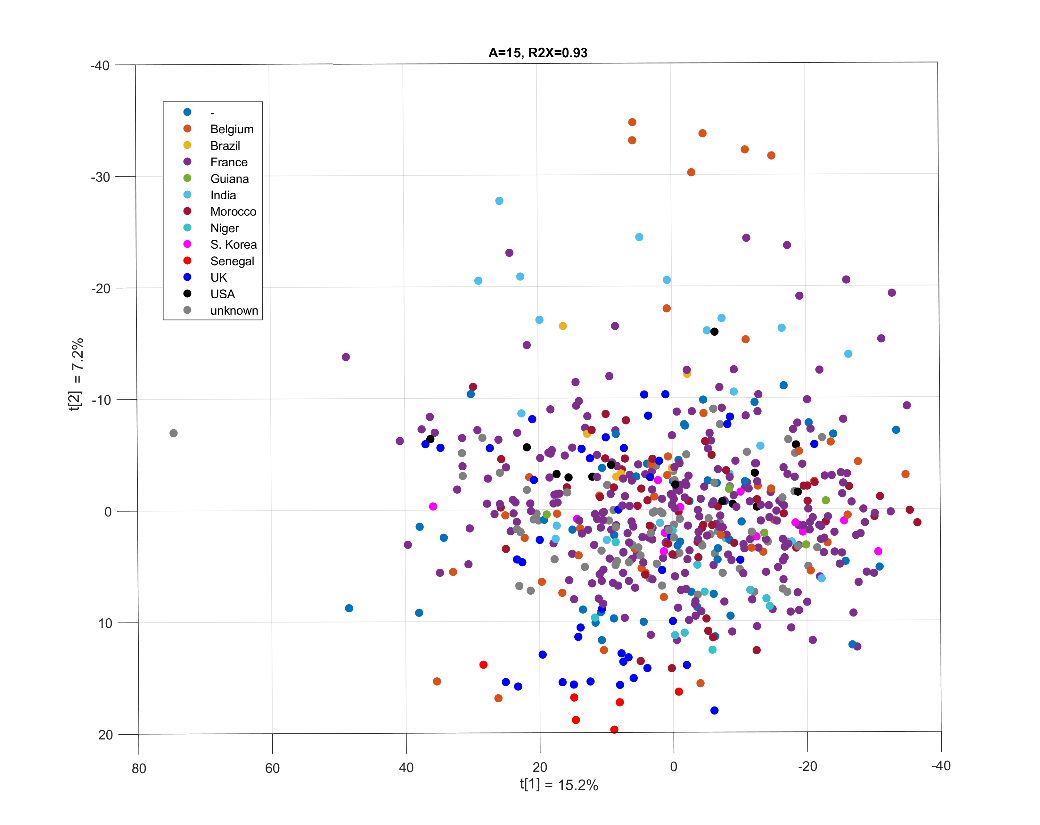  B |

**Supplemental Figure 3.** PCA scatter plots of source and country of origin. Data points correspond to isolate sample replicates colored by source (A) and by country of origin (B).


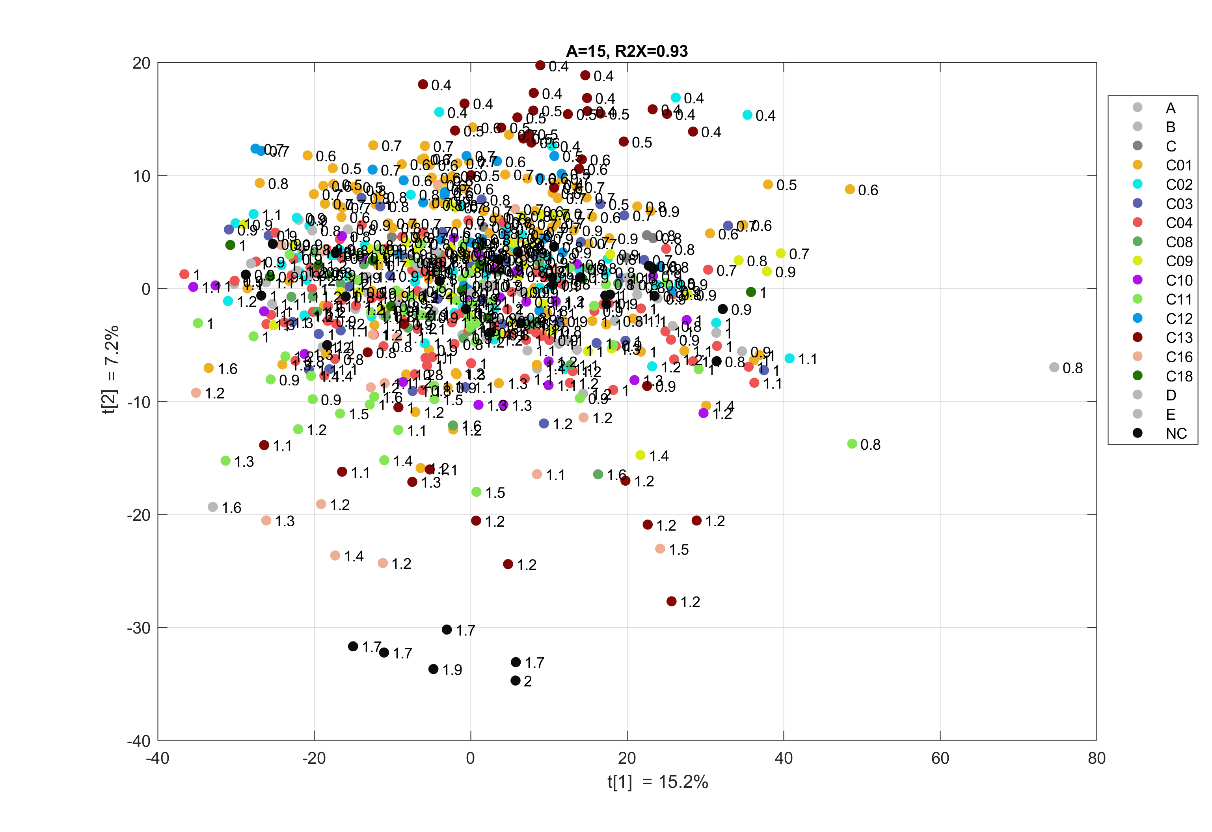


OD

**Supplemental Figure 4. Metabolic fingerprint PCA scatter plot with data points corresponding to isolate sample replicates labelled by optical density (OD) and colored by clade.** Green triangle shows the OD values, with the base indicating higher OD values and the apex lower OD values.

| Metabolic fingerprint | Quantified metabolites | VIP > 1 |
| --- | --- | --- |
| Clade 01 vs Clade 02 |  |  |
| 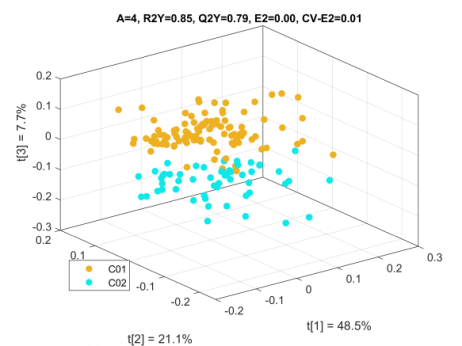 | 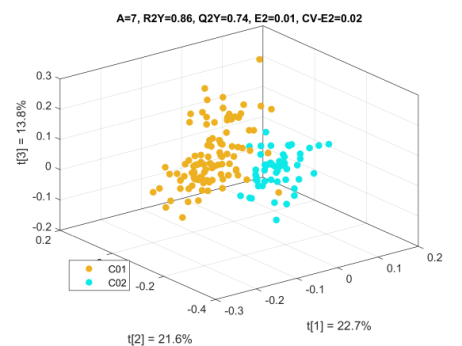 | Guanosine, Adenosine, Threonine, Asparagine, Trehalose, Pyruvate, Choline, sn-Glycero-3-phosphocholine, Succinate, Serine, Proline, D-(+)-malic acid |
| Clade 01 vs Clade 03 |  |  |
| 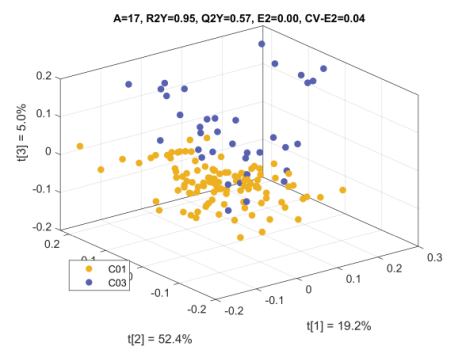 | 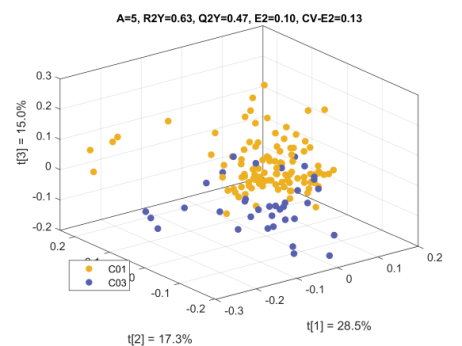 | Fumarate, Guanosine, Glycerol, Acetate, Adenosine, Glucose, Betaine, D-(+)-malic acid, Ornithine, Trehalose, Valine, Pyruvate, Asparagine |
| Clade 01 vs Clade 04 |  |  |
| 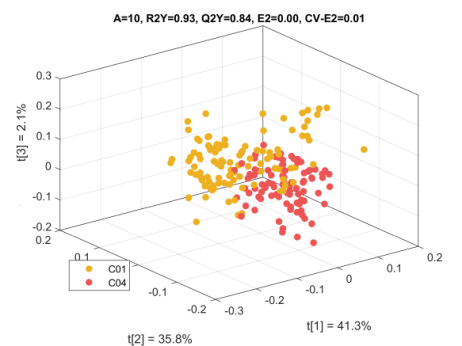 | 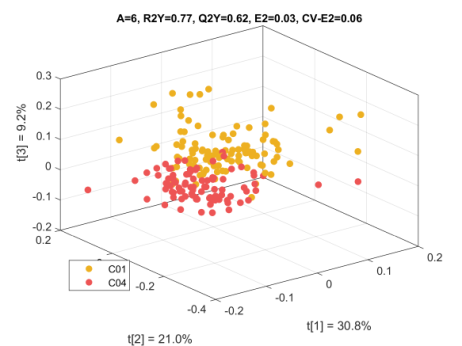 | Guanosine, Acetate, Glycerol, Uridine, Adenosine, Glucose, Formate, Succinate, Threonine, Fumarate, Trehalose, Niacinamide, sn-Glycero-3-phosphocholine, Pyruvate |
| Clade 01 vs Clade 09 |  |  |
| 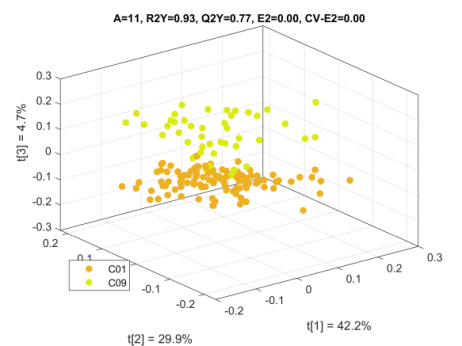 | 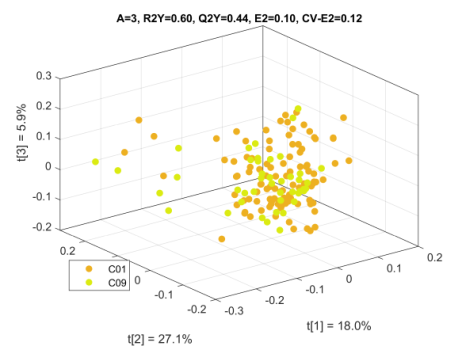 | Guanosine, Trehalose, Nicotinate, Niacinamide, sn-Glycero-3-phosphocholine, Formate, Threonine, Uridine, Glycerol, Glucose |
| Clade 01 vs Clade 11 |  |  |
| 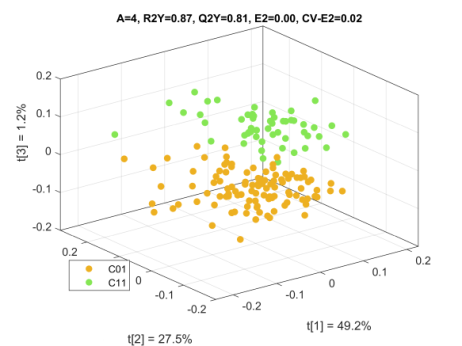 | 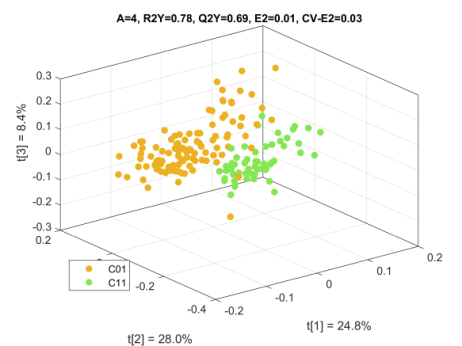 | Guanosine, sn-Glycero-3-phosphocholine, Choline, Pyruvate, Trehalose, Glucose, Uridine, Succinate, Adenosine, Threonine, Aspartate |
| Clade 01 vs Clade 13 |  |  |
| 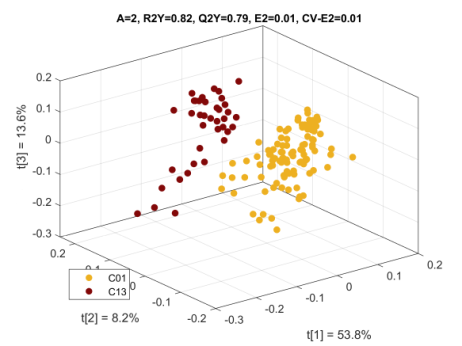 | 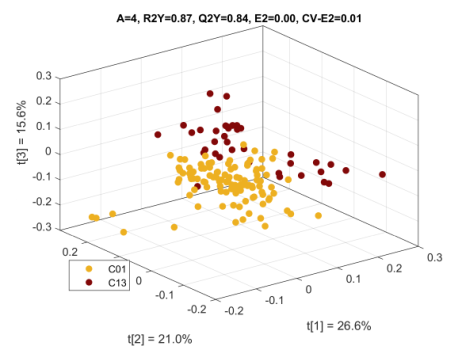 | Choline, Trehalose, Fumarate, Glycerol, D-(+)-malic acid, Formate, Nicotinate, Uridine, Acetate, sn-Glycero-3-phosphocholine, Asparagine |
| Clade 02 vs Clade 03 |  |  |
| 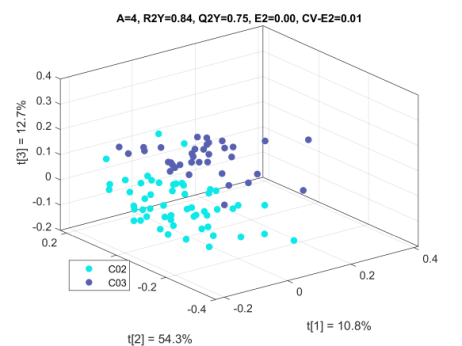 | 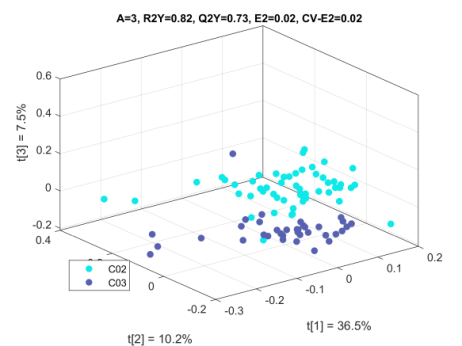 | Fumarate, Adenosine, Threonine, Succinate, Asparagine, Choline, Serine, Glucose, Acetate, Ornithine, Proline |
| Clade 02 vs Clade 04 |  |  |
| 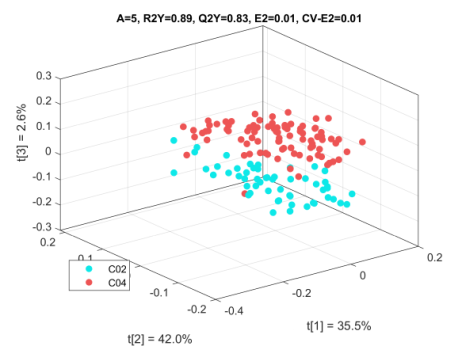 | 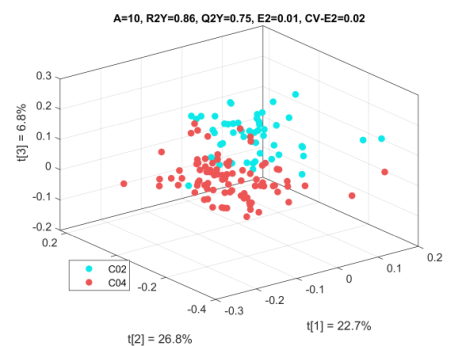 | Adenosine, Acetate, Guanosine, Pyruvate, Glucose, Choline, Formate, Fumarate, Asparagine, Threonine, Uridine, sn-Glycero-3-phosphocholine |
| Clade 02 vs Clade 09 |  |  |
| 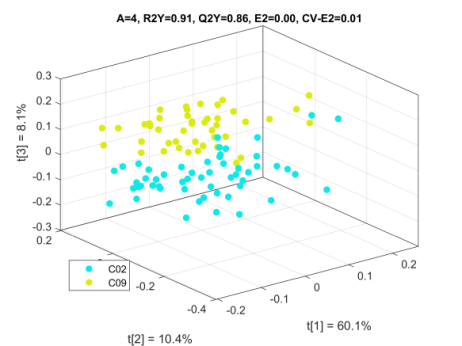 | 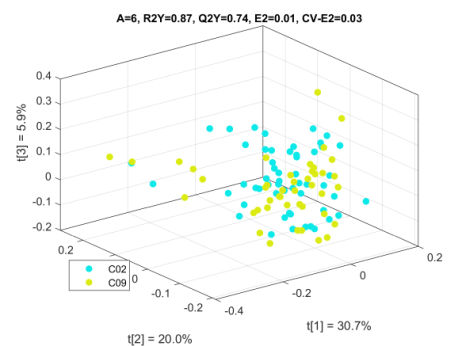 | Adenosine, Formate, Choline, Nicotinate, Pyruvate, Asparagine, Succinate, Glycerol, Threonine, Fumarate, Uracil |
| Clade 02 vs Clade 11 |  |  |
| 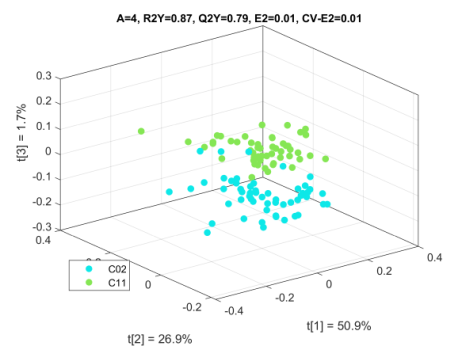 | 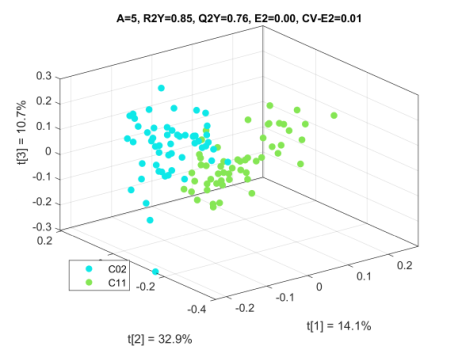 | Adenosine, Guanosine, Glucose, Proline, Choline, Ethanol, Niacinamide, Formate, Acetate, Asparagine, Uridine, Threonine |
| Clade 02 vs Clade 13 |  |  |
| 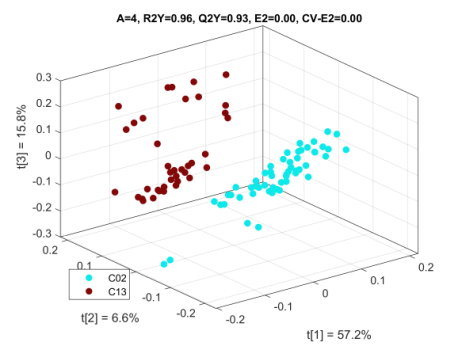 | 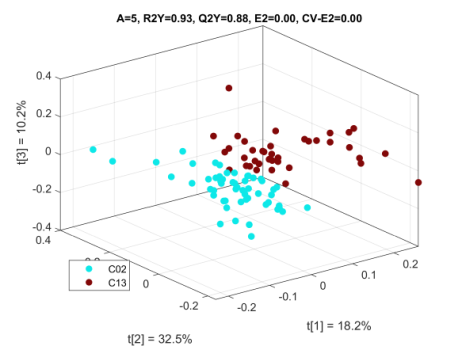 | Trehalose, Choline, Fumarate, Nicotinate, D-(+)-malic acid, Acetate, Proline, Formate, Guanosine, Pyruvate |
| Clade 03 vs Clade 04 |  |  |
| 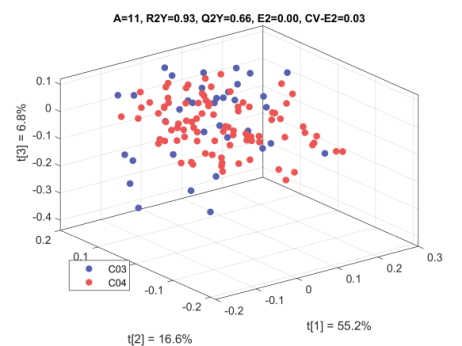 | 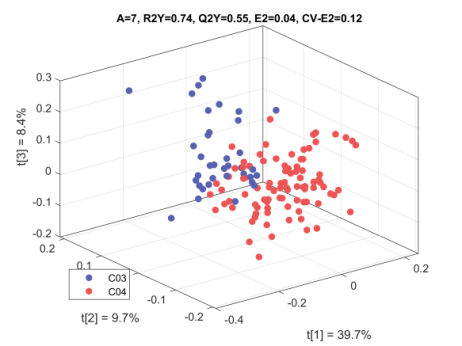 | Fumarate, Guanosine, Trehalose, Succinate, Adenosine, Isoleucine, Arginine, Formate, Threonine, Acetate, Asparagine, Betaine |
| Clade 03 vs Clade 09 |  |  |
| 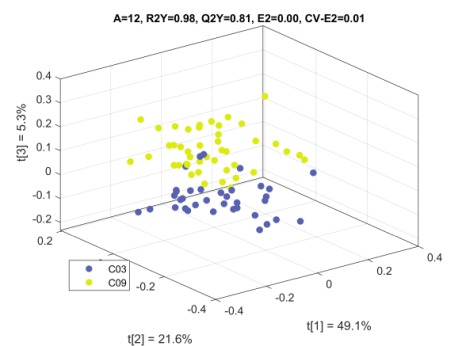 | 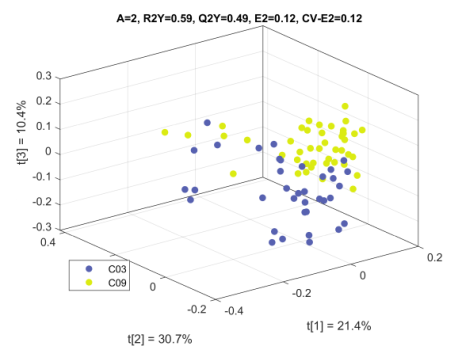 | Formate, Glycerol, Fumarate, Trehalose, Nicotinate, Betaine, Acetate, Niacinamide, Glucose, Glutamate |
| Clade 03 vs Clade 11 |  |  |
| 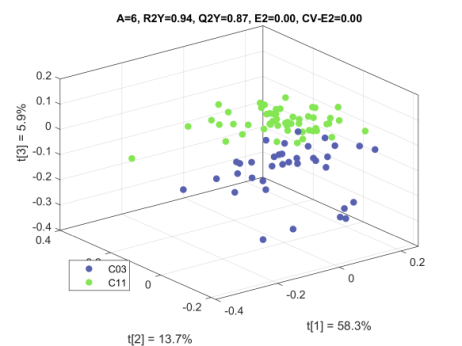 | 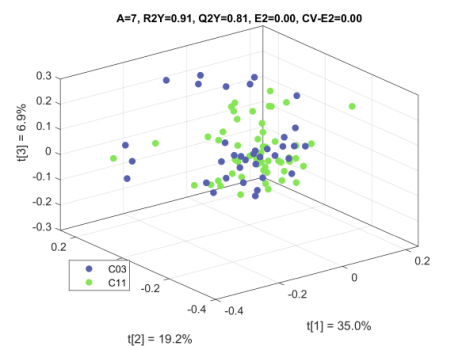 | Choline, Succinate, Fumarate, Trehalose, Guanosine, Adenosine, Acetate, Asparagine, sn-Glycero-3-phosphocholine, Lysine, Threonine, Ornithine, Arginine, Ethanol, Pyroglutamate, Glucose |
| Clade 03 vs Clade 13 |  |  |
| 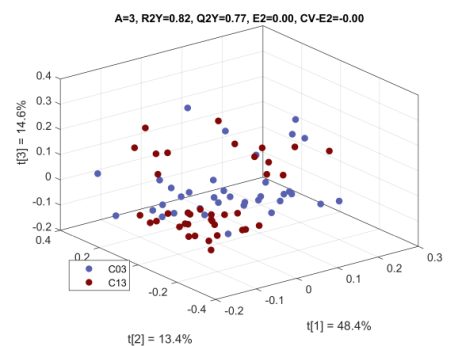 | 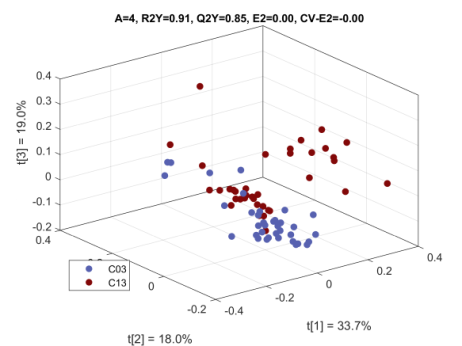 | Trehalose, Choline, Acetate, Nicotinate, Guanosine, Formate, Fumarate, Niacinamide, Betaine, Proline |
| Clade 04 vs Clade 09 |  |  |
| 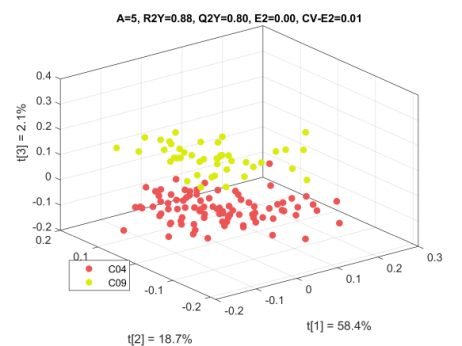 | 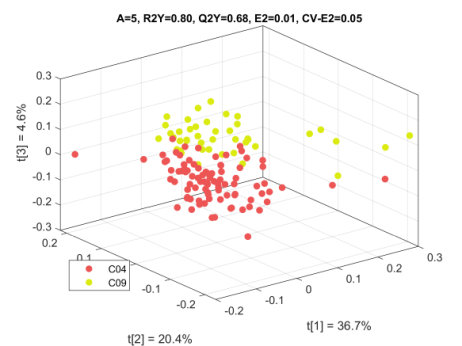 | Glycerol, Acetate, Formate, Pyruvate, Nicotinate, Guanosine, Fumarate, Adenosine, Succinate, Glucose |
| Clade 04 vs Clade 11 |  |  |
| 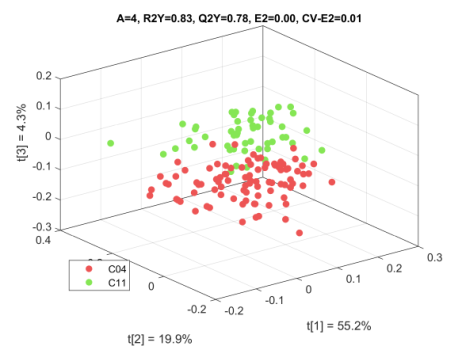 | 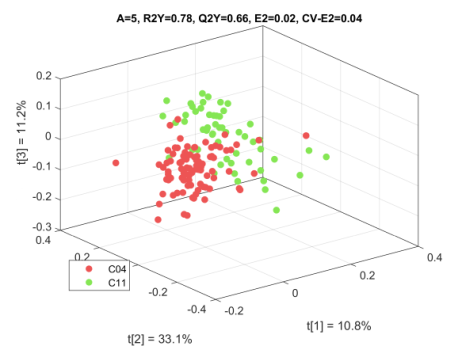 | Acetate, Choline, Formate, Proline, Trehalose, Ethanol, Pyruvate, Glucose, Fumarate, sn-Glycero-3-phosphocholine, Phenylalanine, Niacinamide |
| Clade 04 vs Clade 13 |  |  |
| 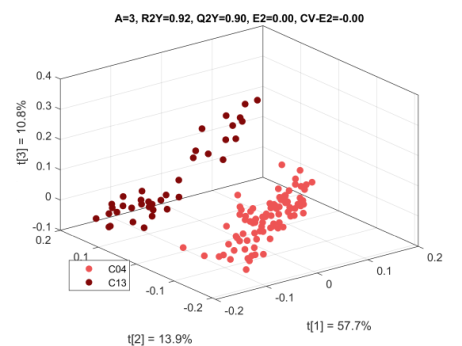 | 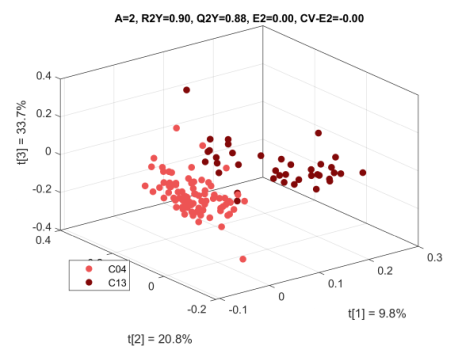 | Trehalose, Choline, Acetate, Fumarate, Guanosine, Nicotinate, D-(+)-malic acid, Proline, Glycerol, Formate |
| Clade 09 vs Clade 11 |  |  |
| 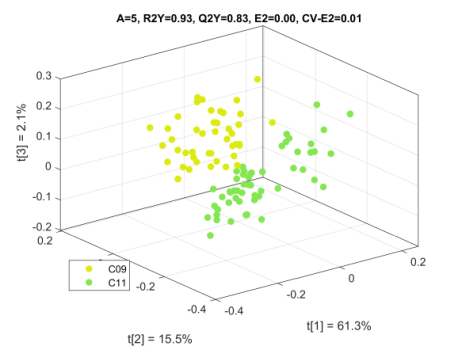 | 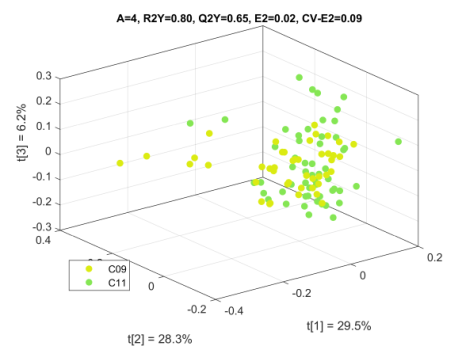 | Choline, Guanosine, Nicotinate, Glucose, Adenosine, Pyruvate, Formate, Glycerol, Succinate, Niacinamide, Acetate, Glutamate |
| Clade 09 vs Clade 13 |  |  |
| 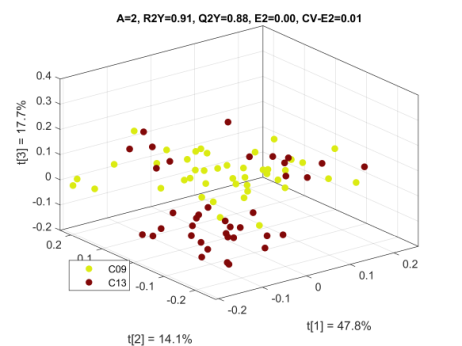 | 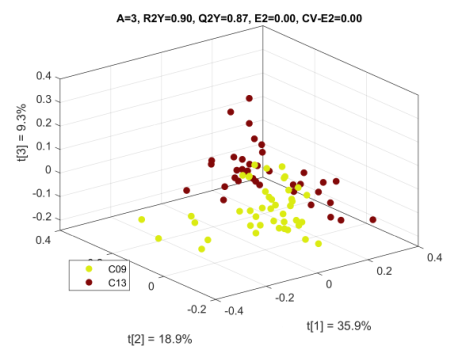 | Trehalose, Choline, Glycerol, Fumarate, Formate, Acetate, D-(+)-malic acid, Guanosine, Glucose |
| Clade 11 vs Clade 13 |  |  |
| 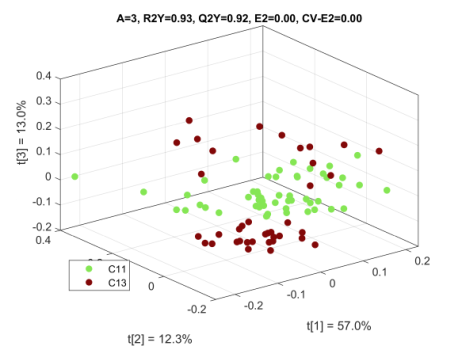 | 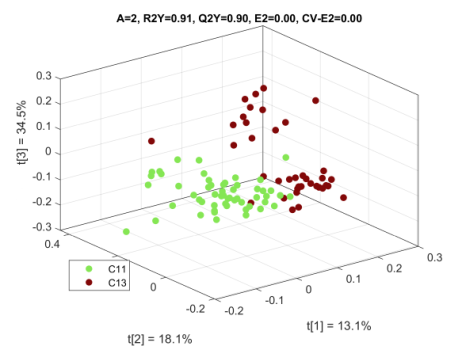 | Choline, Trehalose, Fumarate, Nicotinate, Guanosine, Formate, Glycerol, D-(+)-malic acid, Arginine, Glucose |

**Supplemental Figure 5: Clade pairwise comparison.** Pairwise comparison of metabolic fingerprints and quantified metabolites datasets were done using supervised analysis, PLS DA. For the quantified metabolites, the metabolites with VIP (variable importance in the projection) scores > 1 were listed. Data points correspond to individual isolate sample replicate colored by clade. Plots for the largest clades (Clade 01, 02, 03, 04, 09, 11) and Clade 13 are shown.

| 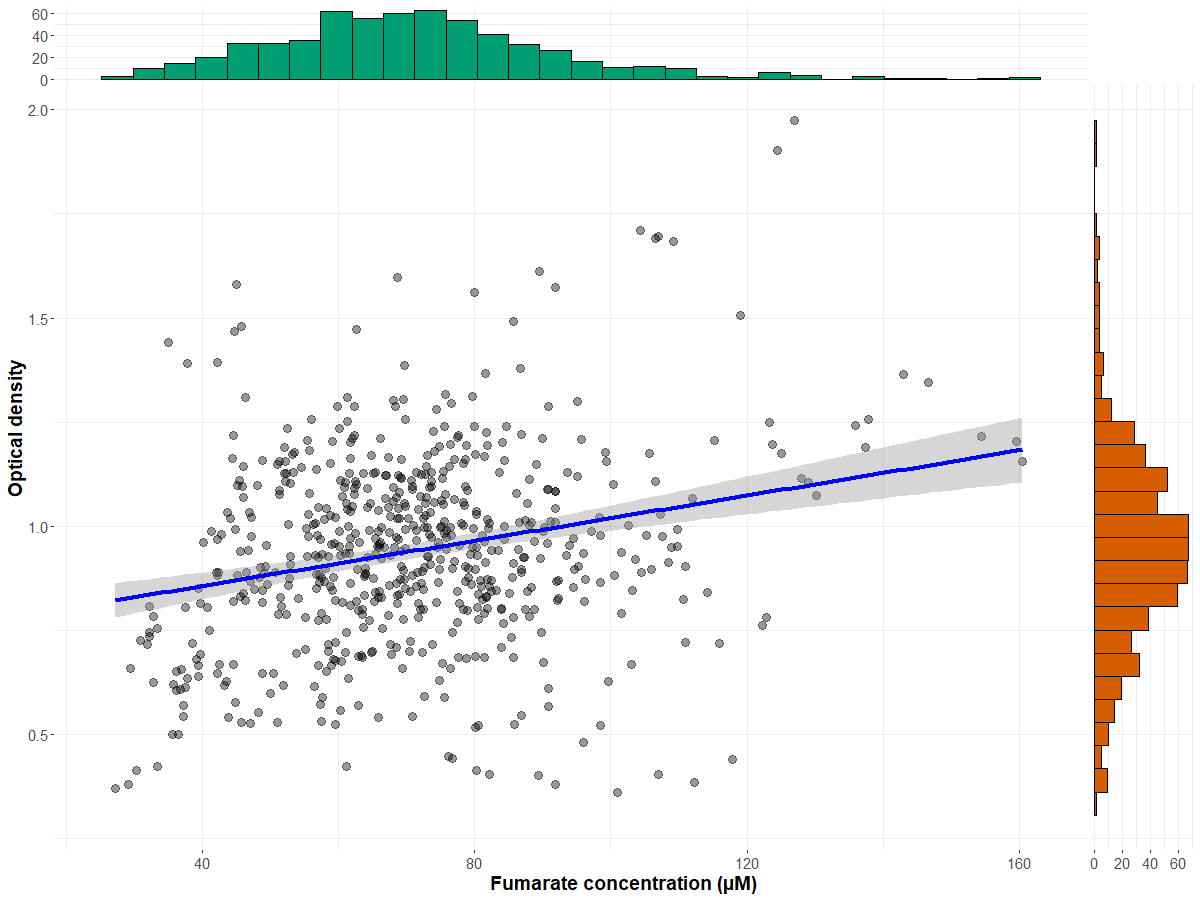  A |
| --- |
| 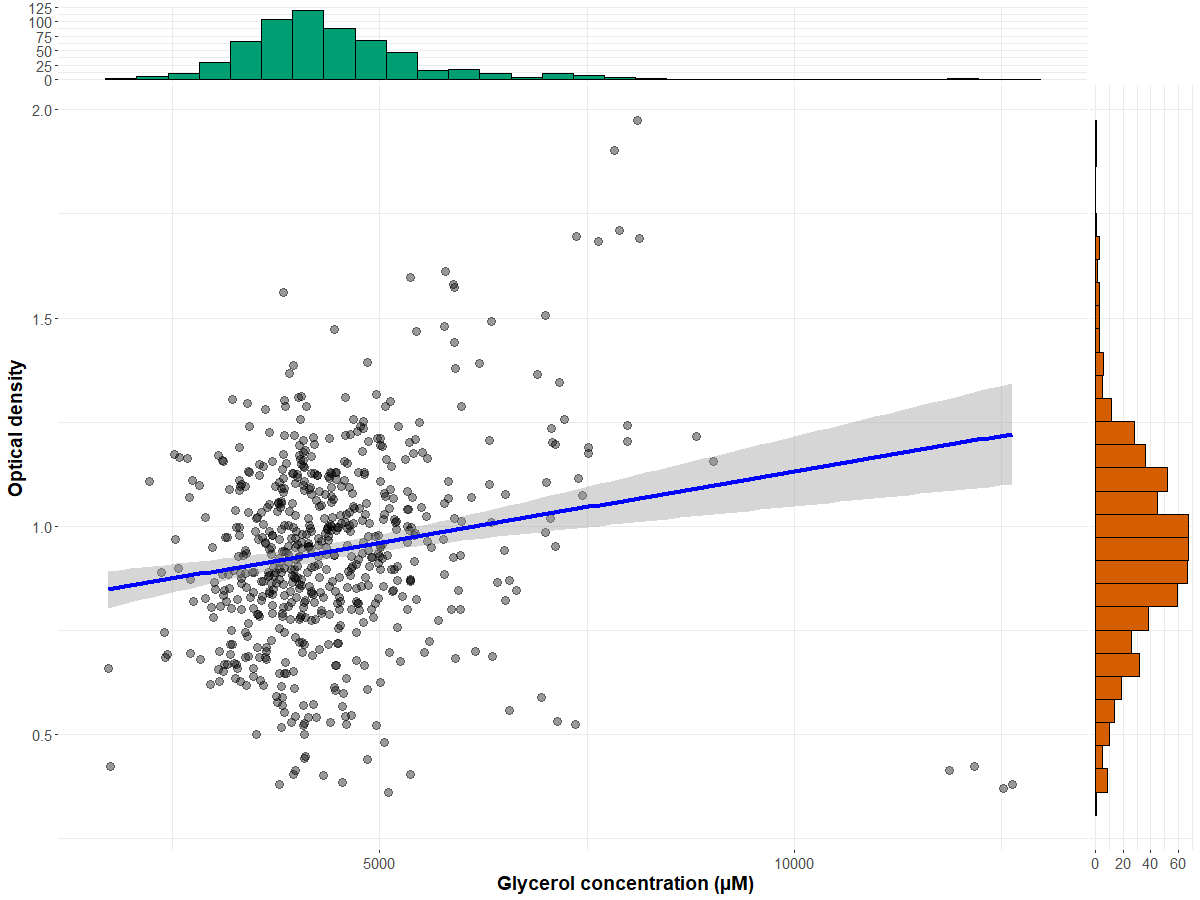  B |

**Supplemental Figure 6. Correlation of metabolites exhibiting highest isolate-specific variation with OD.** Spearman rank correlation between OD and fumarate (A) or glycerol (B) concentrations are shown. Shapiro Wilk analyses for OD (*p*< 0.001), fumarate (*p*< 0.001) and glycerol (*p*< 0.001) were done prior to correlation analysis. The marginal histograms correspond to the distribution of data points (n = 614 pairs) and the smooth line fitted on the scatterplot were generated using default values in the R package ggstatsplot.
